# Convergence of a common solution for broad ebolavirus neutralization by glycan cap-directed human antibodies

**DOI:** 10.1016/j.celrep.2021.108984

**Published:** 2021-04-13

**Authors:** Charles D. Murin, Pavlo Gilchuk, Philipp A. Ilinykh, Kai Huang, Natalia Kuzmina, Xiaoli Shen, Jessica F. Bruhn, Aubrey L. Bryan, Edgar Davidson, Benjamin J. Doranz, Lauren E. Williamson, Jeffrey Copps, Tanwee Alkutkar, Andrew I. Flyak, Alexander Bukreyev, James E. Crowe, Andrew B. Ward

**Affiliations:** 1 Department of Integrative Structural and Computational Biology, The Scripps Research Institute, La Jolla, CA 92037, USA; 2 Vanderbilt Vaccine Center, Vanderbilt University Medical Center, Nashville, TN 37232, USA; 3 Galveston National Laboratory, Galveston, TX 77550, USA; 4 Department of Pathology, University of Texas Medical Branch, Galveston, TX 77555, USA; 5 Laboratory of Genetics and Helmsley Center for Genomic Medicine, The Salk Institute for Biological Sciences, La Jolla, CA 92037, USA; 6 Integral Molecular, Inc., Philadelphia, PA 19104, USA; 7 Department of Pathology, Microbiology and Immunology, Vanderbilt University Medical Center, Nashville, TN 37232, USA; 8 Department of Microbiology & Immunology, University of Texas Medical Branch, Galveston, TX 77555, USA; 9 Department of Pediatrics, Vanderbilt University Medical Center, Nashville, TN 37232, USA; 10 Present address: NanoImaging Services Inc., San Diego, CA 92121, USA; 11 Present address: Division of Biology and Biological Engineering, California Institute of Technology, Pasadena, CA 91125, USA; 12 Lead contact

## Abstract

Antibodies that target the glycan cap epitope on the ebolavirus glycoprotein (GP) are common in the adaptive response of survivors. A subset is known to be broadly neutralizing, but the details of their epitopes and basis for neutralization are not well understood. Here, we present cryoelectron microscopy (cryo-EM) structures of diverse glycan cap antibodies that variably synergize with GP base-binding antibodies. These structures describe a conserved site of vulnerability that anchors the mucin-like domains (MLDs) to the glycan cap, which we call the MLD anchor and cradle. Antibodies that bind to the MLD cradle share common features, including use of *IGHV1–69* and *IGHJ6* germline genes, which exploit hydrophobic residues and form β-hairpin structures to mimic the MLD anchor, disrupt MLD attachment, destabilize GP quaternary structure, and block cleavage events required for receptor binding. Our results provide a molecular basis for ebolavirus neutralization by broadly reactive glycan cap antibodies.

## In brief

A rare subset of ebolavirus antibodies targeting the glycan cap are broadly neutralizing. Murin et al. report cryo-EM structures and custom *in vitro* assays identifying a conserved site of vulnerability in the glycan cap and detail mechanisms of action, including structural mimicry, trimer instability, and blocking cleavage.

## Graphical Abstract

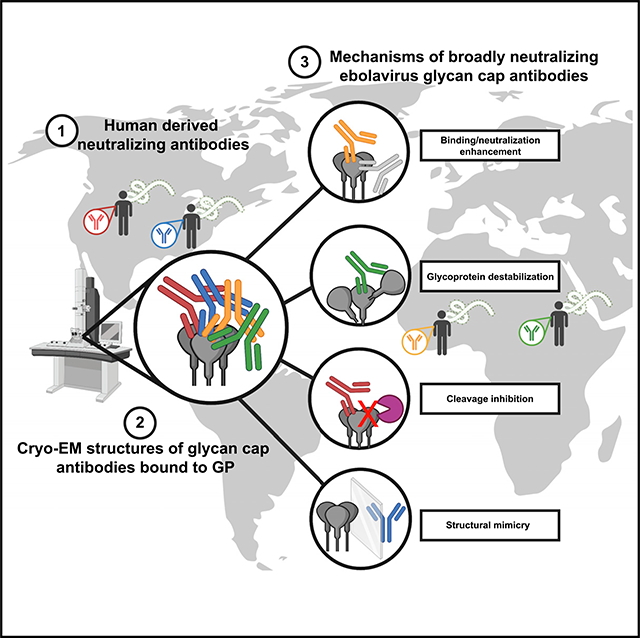

## INTRODUCTION

There is mounting evidence that protection from filovirus infection can be achieved through use of monoclonal antibodies (mAbs) that target the glycoprotein (GP) surface (Bornholdt et al., 2019; Brannan et al., 2019; Mire et al., 2017; Qiu et al., 2014; Saphire et al., 2018b). Several structures of antigen-antibody complexes indicate that antibodies can access nearly any region on the surface of GPs (Flyak et al., 2015, 2018; Gilchuk et al., 2018; Milligan et al., 2019; Murin et al., 2018, 2019; Pallesen et al., 2016; Pascal et al., 2018; Saphire et al., 2018a; Wec et al., 2017; West et al., 2018; Zhao et al., 2017). Such antibodies have utility as post-exposure therapeutic agents when used in combination, such as the tri-mAb cocktail REGN-EB3, which has demonstrated high efficacy in animal models (Pascal et al., 2018) and in a clinical trial carried out during a recent ebolavirus (EBOV) outbreak (Mulangu et al., 2019). REGN-EB3 is only effective against EBOV; however, a pan-EBOV therapeutic agent that recognizes multiple EBOVs that cause severe disease in humans and major outbreaks, including Bundibugyo ebolavirus (BDBV) and Sudan ebolavirus (SUDV), would be ideal, given the unpredictability of EBOV outbreaks.

Cross-reactive antibodies often target regions of conserved sequences vital to viral function, such as the receptor binding site (RBS) (Flyak et al., 2015; Hashiguchi et al., 2015; Howell et al., 2016; King et al., 2018), the internal fusion loop (IFL) (Milligan et al., 2019; Murin et al., 2018; West et al., 2018; Zhao et al., 2017), the base of the GP (Gilchuk et al., 2018; Misasi et al., 2016), and the heptad repeat 2 (HR2) region (Bornholdt et al., 2016b; Flyak et al., 2018). Less conserved regions, such as the glycan cap and mucin-like domain (MLD), can also be targeted by protective antibodies and typically represent the largest antibody responses found in survivors; however, such antibodies are usually weakly or non-neutralizing and species specific (Murin et al., 2014; Zeitlin et al., 2011). For example, the antibody 13C6, which is included in the antibody cocktail ZMapp, targets the glycan cap but is low in potency for viral neutralization and is thought to provide protection by facilitating a superior cellular response (Murin et al., 2014; Pallesen et al., 2016). Furthermore, the glycan cap/head epitope in the trimeric membrane form of the GP is also partially present on sGP, the soluble dimer of the GP that is secreted in abundance during natural infection (Cook and Lee, 2013; de La Vega et al., 2015; Pallesen et al., 2016). Finally, the GP is remodeled massively during endosomal entry in processes mediated by host proteases, during which the glycan cap and MLD are removed (Bornholdt et al., 2016a; Lee and Saphire, 2009). Nevertheless, several antibodies have been identified that bind within the glycan cap and potently neutralize EBOV, BDBV, and SUDV (Bornholdt et al., 2016b; Flyak et al., 2016; Gilchuk et al., 2020; Pascal et al., 2018; Saphire et al., 2018a). The mechanistic basis for this activity, however, is not well explored.

We previously characterized pan-EBOV-neutralizing mAbs isolated from a survivor cohort of the EBOV 2013–2016 outbreak (Gilchuk et al., 2018, 2020). Several antibodies that recognize the glycan cap revealed synergistic activity for GP binding and virus neutralization when paired with GP base-binding antibodies. One such pair, EBOV-548 and EBOV-520, revealed enhanced activity in the cocktail compared with treatment activity by either antibody alone. Structural evaluation revealed that EBOV-520 recognized the 3^10^ pocket, which is partially shielded by the β17-β18 loop in uncleaved GP. EBOV-548, which binds to the glycan cap, removed this steric hindrance by dislodging and mimicking the β18-β18′ hairpin obscuring the 3^10^ pocket. These data reveal a structural mechanism for synergy mediated by a glycan cap-directed antibody.

We sought to determine whether glycan cap antibodies from other survivors use similar mechanisms of protection and synergy as the EBOV-548/EBOV-520 combination (Bramble et al., 2018; Flyak et al., 2015, 2016, 2018; Gilchuk et al., 2018). This collection of antibodies, including two mAbs from a newly described survivor cohort, was tested for the ability to potentiate the activity of the GP base-binding, broadly neutralizing antibodies EBOV-520 and EBOV-515 (Gilchuk et al., 2020). Additionally, we observed and quantified antibody-induced GP trimer instability. Subsequent analysis by cryoelectron microscopy (cryo-EM) revealed a conserved structural motif, similar to that found in EBOV-548, wherein a complementarity-determining region (CDR) exhibited molecular mimicry of the β18-β18′ hairpin in the GP. Finally, we also assessed the ability of glycan cap antibodies to block GP cleavage events necessary for RBS exposure. Our data provide evidence of a mechanism behind the activity of broadly neutralizing and synergistic glycan cap antibodies to EBOVs and suggest a rational strategy for design of therapeutic antibody cocktails.

## RESULTS

### Glycan cap antibody synergy is a common feature and is associated with GP instability

We previously described an assay to determine glycan cap antibody-based synergy of the GP base-binding antibodies EBOV-515 and EBOV-520 (Gilchuk et al., 2020). Here, we extended this assay to glycan cap antibodies from other survivor cohorts. We chose previously isolated antibodies based on properties similar to the synergistic glycan cap mAb EBOV-548, including (1) synergy with EBOV-520 and/or EBOV-515, (2) broad reactivity and neutralization, (3) long CDRH3 loops, (4) cross-reactivity with sGP, and/or (5) protection *in vivo*. Based on these criteria, we chose the following antibodies: BDBV-43, BDBV-329, BDBV-289, EBOV-442, EBOV-437, and EBOV-237 (Flyak et al., 2016; Gilchuk et al., 2018; Qiu et al., 2012; Williamson et al., 2019; Wilson et al., 2000). EBOV-548, 13C6, and an unrelated human mAb directed to dengue virus (DENV) envelope protein, 2D22 (Fibriansah et al., 2015), were included for comparative purposes and as controls (Table S1). In addition, we also tested two new antibodies, EBOV-293 and EBOV-296, which we isolated from an individual treated for EBOV infection in the United States (Table S1; Figure S1; STAR Methods). Ten characterized glycan cap antibodies potently bound to sGP, as judged by the half-maximal effective concentration (EC_50_), and revealed diverse GP reactivity and virus neutralization profiles and diverse protective efficacy in an EBOV challenge mouse model (Table S1; Figure S1A). In addition, epitope mapping by alanine scanning mutagenesis library analysis identified key contact residues for each antibody (Table S1; Figure S1B). Furthermore, several of these antibodies have exceptionally long CDRH3 loops, such as the 33-amino-acid loop of BDBV-329 (Table S2).

**Figure 1.**
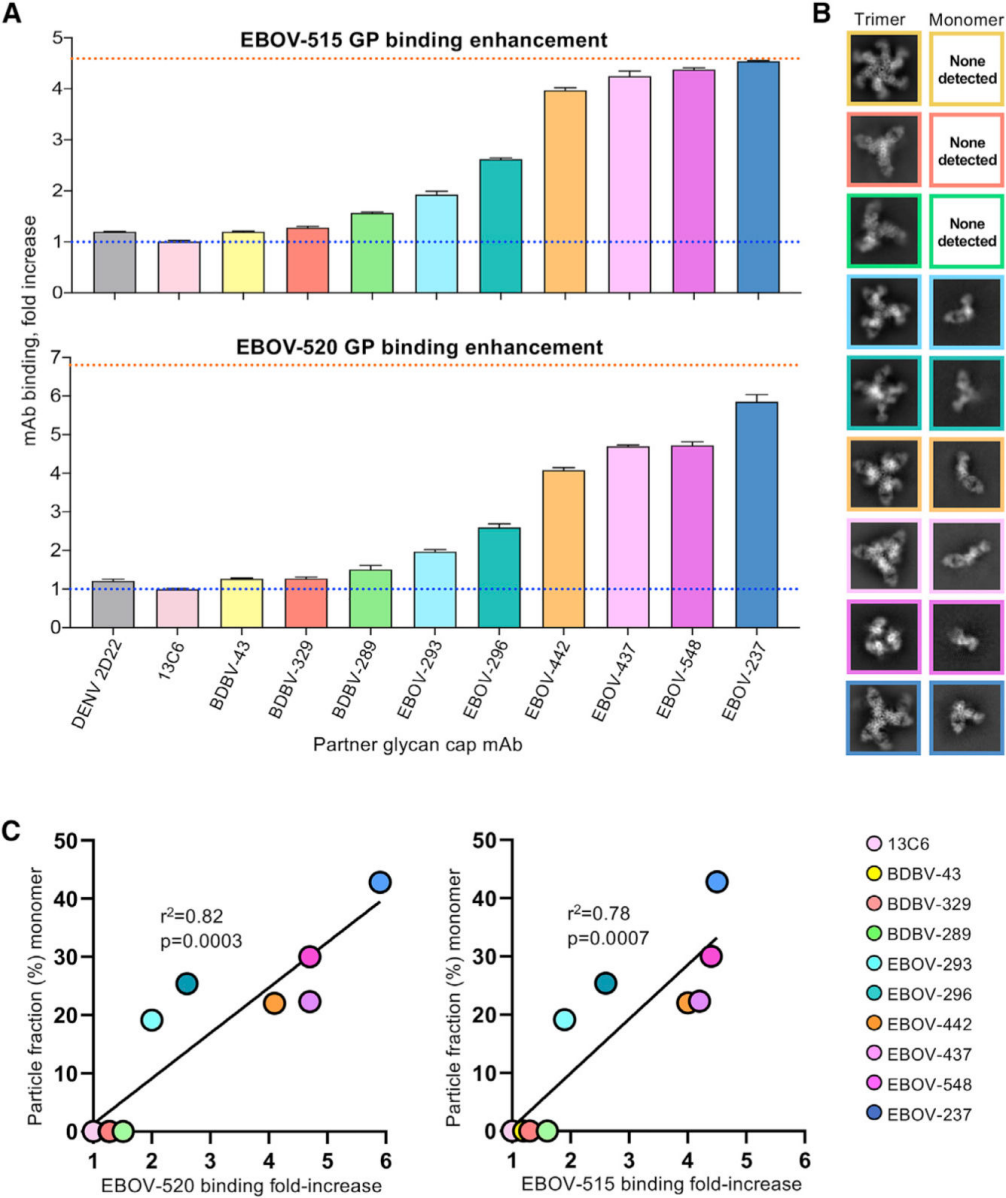
Glycan cap antibody synergy and GP destabilization (A) Jurkat cell surface-displayed EBOV GP binding was assessed using fluorescently labeled EBOV-515 or EBOV-520 after prior incubation of cells with individual unlabeled glycan cap antibodies. The blue dotted line represents basal binding of base antibodies without glycan cap antibodies. The orange dotted line represents maximal binding of base antibodies to GP_CL_. Data are shown as mean ± SD of technical triplicates. (B) Negative-stain 2D-class averages of GP complexes bound to glycan cap antibodies and EBOV-515, demonstrating examples of intact trimeric complexes (left) and monomeric complexes (right). (C) Correlation analysis of antibody synergy and GP destabilization by glycan cap antibodies. Curve fitting was performed using simple linear regression analysis. The relationship between the two variables was determined using Pierson correlation analysis. r^2^ quantifies goodness of fit, and the p value indicates whether the slope is significantly non-zero. See also Tables S1 and S2.

We then analyzed all 10 glycan cap mAbs for binding enhancement of the base-region-directed mAbs EBOV-515 or EBOV-520 using an approach described previously (Gilchuk et al., 2020). Synergy for each glycan cap antibody followed similar patterns for EBOV-515 and EBOV-520, although enhancement of EBOV-520 binding appears to be higher, likely because of differences in the molecular nature of the epitope (Figure 1A). A steady range of synergistic patterns from no enhancement (for 13C6) to binding nearly equivalently to cleaved GP (GP_CL_) (for EBOV-237) were observed (Figure 1A). BDBV-329 and EBOV-237 are monospecific for the autologous viruses BDBV and EBOV, respectively (Table S1).

In several of our 2D classes of glycan cap antibody complexes, we noticed that the GP trimer fell apart into GP monomers, similar to what we had observed with our previous characterization of EBOV-548 (Gilchuk et al., 2020). The amount of GP trimer destabilization was variable across all complexes, with some antibodies inducing a large amount of GP monomers and others maintaining a stable GP trimer. We specifically avoid inclusion of monomers during protein purification for cryo-EM to obtain a pure fraction of trimeric GP as starting material. We therefore hypothesized that glycan cap antibodies destabilize trimers, which, in turn, may contribute to their synergistic ability with base-region-binding antibodies.

To quantify GP destabilization, we analyzed cryo-EM data collected for glycan cap antibody complexes (see below). We also included data collected previously for EBOV-548 complexed with GP (Gilchuk et al., 2020). Particles were selected using a difference of Gaussians approach that would not distinguish trimeric complexes from monomeric ones, and then we performed reference-free 2D classification (Figure 1B). All trimeric and monomeric particles were subsequently subclassified, and particle counts were used to determine the percentage of monomeric particles in the cleaned stack of total particles for each dataset.

When plotting the proportion of monomers formed in the presence of each glycan cap antibody, we noted that the amount of destabilization correlated positively with the extent of antibody synergy (Figure 1C). Antibodies that did not synergize with base-region-binding antibodies displayed little to no destabilization, such as 13C6, BDBV-43, BDBV-329, and BDBV-289. As synergy increased, we saw increasing amounts of destabilized trimers, with EBOV-237 demonstrating the highest level of destabilization (Figure 1C).

### Conservation of a structural β-hairpin motif across synergistic glycan cap antibodies

**Figure 2.**
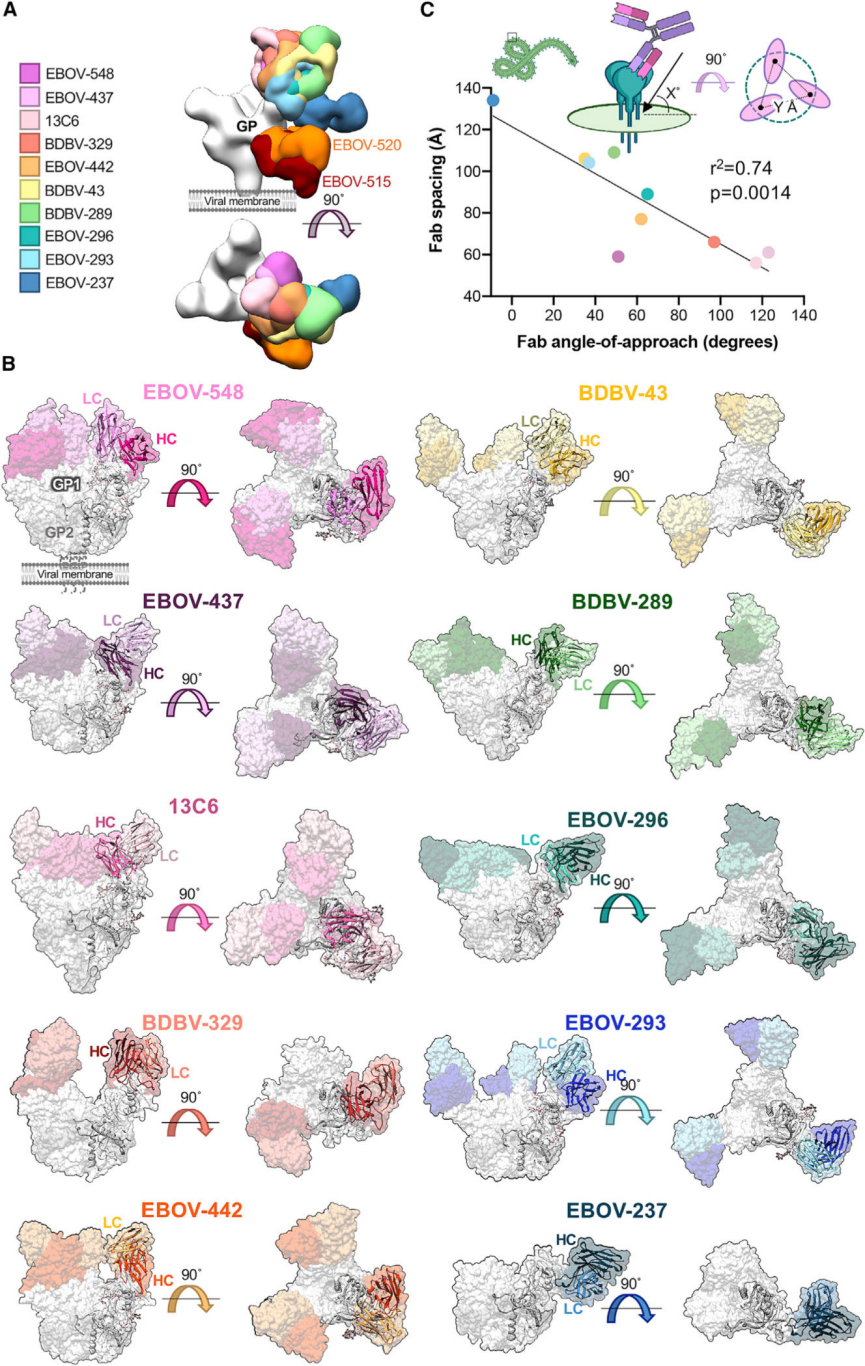
Neutralizing and synergistic glycan cap antibodies bind GP across a wide range of orientations (A) Low-pass-filtered glycan cap Fabs from cryo-EM structures solved in this study as well as elsewhere, bound to EBOV GPΔMuc, are overlaid to compare binding epitope and angle of approach. (B) Surface representations of cryo-EM structures solved in this study with a fitted ribbon model protomer. Shown are side (left) or top (right) views with respect tothe viral membrane. Fab HC is colored in dark tones and LC in light tones. Co-binding antibodies were removed from reconstructions for clarity. (C) Relationship between antibody angle of approach and Fab spacing. An angle of approach of 0° is considered parallel and 90° is considered perpendicular to the viral surface. An angle of approach greater than 90° indicates antibodies that bind inward toward the head domain, whereas less than 0° indicates antibodies that bind upward from the viral membrane. Fab spacing is determined by averaging the distance from the same point on modeled Fab hinge terminal residues in the HC and LC. Antibodies are labeled as in (A). Curve fitting was performed using simple linear regression analysis. The relationship between the two variables was determined using Pierson correlation analysis. r^2^ quantifies goodness of fit, and the p value indicates whether the slope is significantly non-zero. See also Figures S2 and S4.

To determine the structural basis of neutralization and synergy behind glycan cap antibody-based enhancement of base-region-binding antibodies, we solved eight structures of glycan cap antibodies in complex with mucin deleted (ΔMuc) EBOV GP (Makona) and BDBV GPΔMuc (Figures 2A and 2B; Figure S2; Table S3). Antibodies exhibited a range of angles of approach to the GP, from obtuse, such as EBOV-437, to nearly parallel to the viral surface, like EBOV-237 (Figure 2C). Additionally, the antibodies are spaced across the surface of the GP inversely related to their angle of approach (Figure 2C). The angle affects how well these antibodies can access the glycan cap epitope and their binding stoichiometry and could alter Fc presentation and subsequently influence effector function, although this would need to be investigated further.

**Figure 3.**
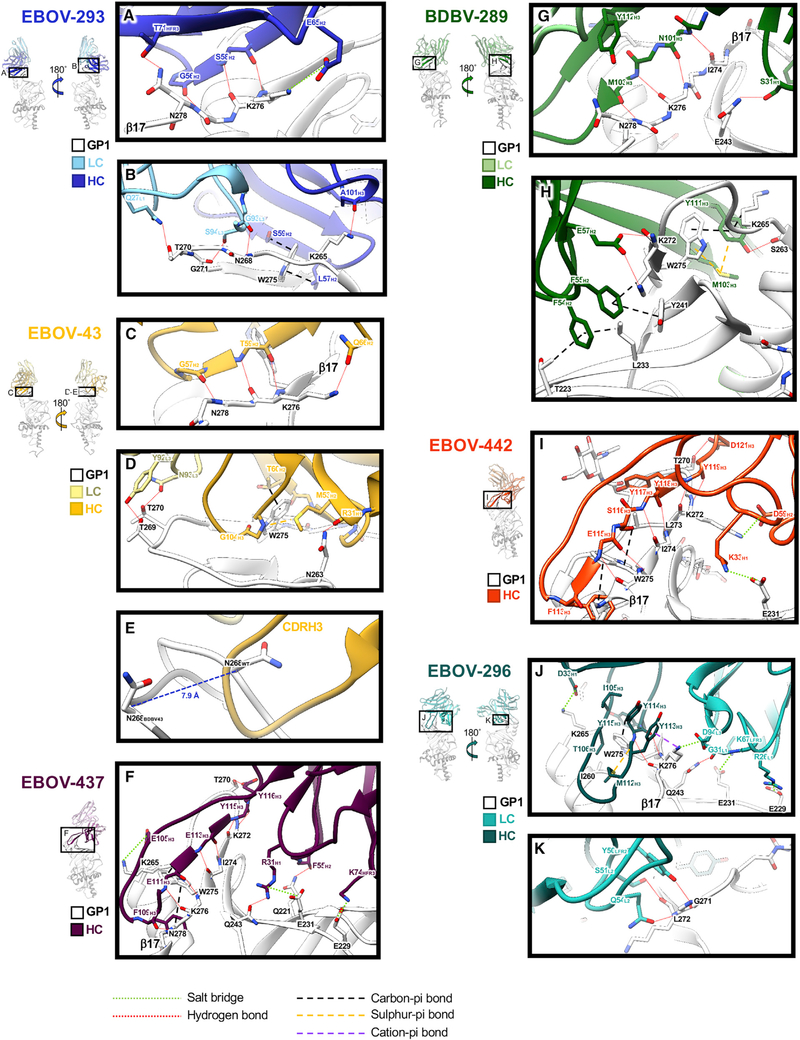
Structural details of glycan cap antibody binding to the GP Single protomers from structural models are shown with close-up views of interacting regions. HCs are rendered in darker colors and LCs in lighter colors, with GP1 colored white. Important residues that coordinate interaction and binding are highlighted. (A) Key residues in the EBOV-293 CDRH2 hydrogen bond along the length of β17 with an additional potential salt bridge between E65_H2_ and K276_GP1_. (B) EBOV-293 CDRH2 and CDRH3 make additional contacts, including at W275, and the LC forms potential hydrogen bonds between α2 and β17. (C) Similar to EBOV-293, the BDBV-43 CDRH2 loop binds along β17. (D) BDBV-43 CDRH2 makes additional contacts at W275 and also contacts the loop between α2 and β17 via its LC. (E) The EBOV-43 CDRH3 loop displaces the loop between α2 and β17, shifting N268 by ~8 Å (apo-GP in white and BDBV-43 bound GP in gray). (F) EBOV-437 makes contact with GP exclusively with its HC, hydrogen bonding along β17 with its CDRH3 and contacting the head domain in several places. (G) BDBV-289 makes extensive hydrogen bonds with its CDRH3 along β17. (H) BDBV-289 CDRH3 contacts W275 via methionine-aromatic and pi-pi interactions. Additional contacts are made with the head domain of GP via hydrophobic interactions with CDRH2. (I) BDBV-442 makes contact with GP exclusively with its HC. CDRH3 makes hydrogen bonds along β17, with W275 with hydrophobic interactions and along the loop between α2 and β17. (J) EBOV-296 binds to the GP along β17, contacting W275 via methionine-aromatic and pi-pi interactions. The LC makes further contact with the head domain of the GP with several potential salt bridges. (K) The EBOV-296 LC also makes contact with the loop between α2 and β17. See also Figures S3 and S4 and Tables S3 and S4.

The resolutions achieved for glycan cap antibody cryo-EM structures ranged from 3.3–4.4 Å for six of our complexes (Table S3; Figures S2A–S2F); however, preferred orientation, sub-stoichiometric antibody fragment (Fab) binding, and trimer instability resulted in limited resolution for BDBV-329- and EBOV-237-bound structures (Figures S2G and S2H). We did, however, model BDBV-329, where resolutions ranged from 4–5 Å at the antibody binding interface (Figure S2G). The local resolutions for the EBOV-237 structure were particularly poor, and we therefore chose only to dock a homology model for interpretation (Figure S2H). Most of our structures were determined in complex with EBOV-515 to assist with angular sampling and alignment, but we chose not to model EBOV-515 and removed this density from our figures for clarity and to focus on glycan cap antibodies (Figures 3A and 3B).

All glycan cap antibodies make contacts exclusively within GP1 and are heavily biased toward heavy-chain (HC) contacts (Figure 3; Table S4; Figures S3 and S4). Antibody contacts are focused on the β17 strand of GP1 from residues 268–280, with the majority of contacts centered around W275 (Figure 3; Figure S4; Table S4), which, when mutated to alanine, abrogates binding (Figure S1B; Table S1). Additionally, most glycan cap antibodies make some contact with the inner head domain (Figure 3; Figure S3; Table S4). These contacts are characterized by hydrogen bonding along the length of β17, with short CDRH2 loops for EBOV-293 (Figures 3A and 3B; Figure S3A) and BDBV-43 (Figures 3C and 3D; Figure S3B) or extended, long (≥21 amino acids) CDRH3 loops for EBOV-437 (Figure 3F; Figure S3C), BDBV-289 (Figures 3G and 3H; Figure S3D), EBOV-442 (Figure 3I; Figure S3E), and EBOV-296 (Figures 3J and 3K; Figure S3F), very similar to EBOV-548, as we reported previously (Gilchuk et al., 2020). Outside of the hydrogen bonding that occurs along β17, several glycan cap antibodies make additional stabilizing bonds, including hydrogen bonds, salt bridges, and carbon-pi and pi-pi bonds with other portions of GP1 (Figure 3). Methionine-aromatic interactions also appear in several of the glycan cap antibodies, particularly with W275 in GP1 (Figures 3D, 3H, and 3J). These types of interactions are thought to provide additional stability compared with purely hydrophobic interactions, can act at long distances (~5–6 Å), and are thought to be less sensitive to changes in the local environment (Valley et al., 2012), which may contribute to the increased breadth.

**Figure 4.**
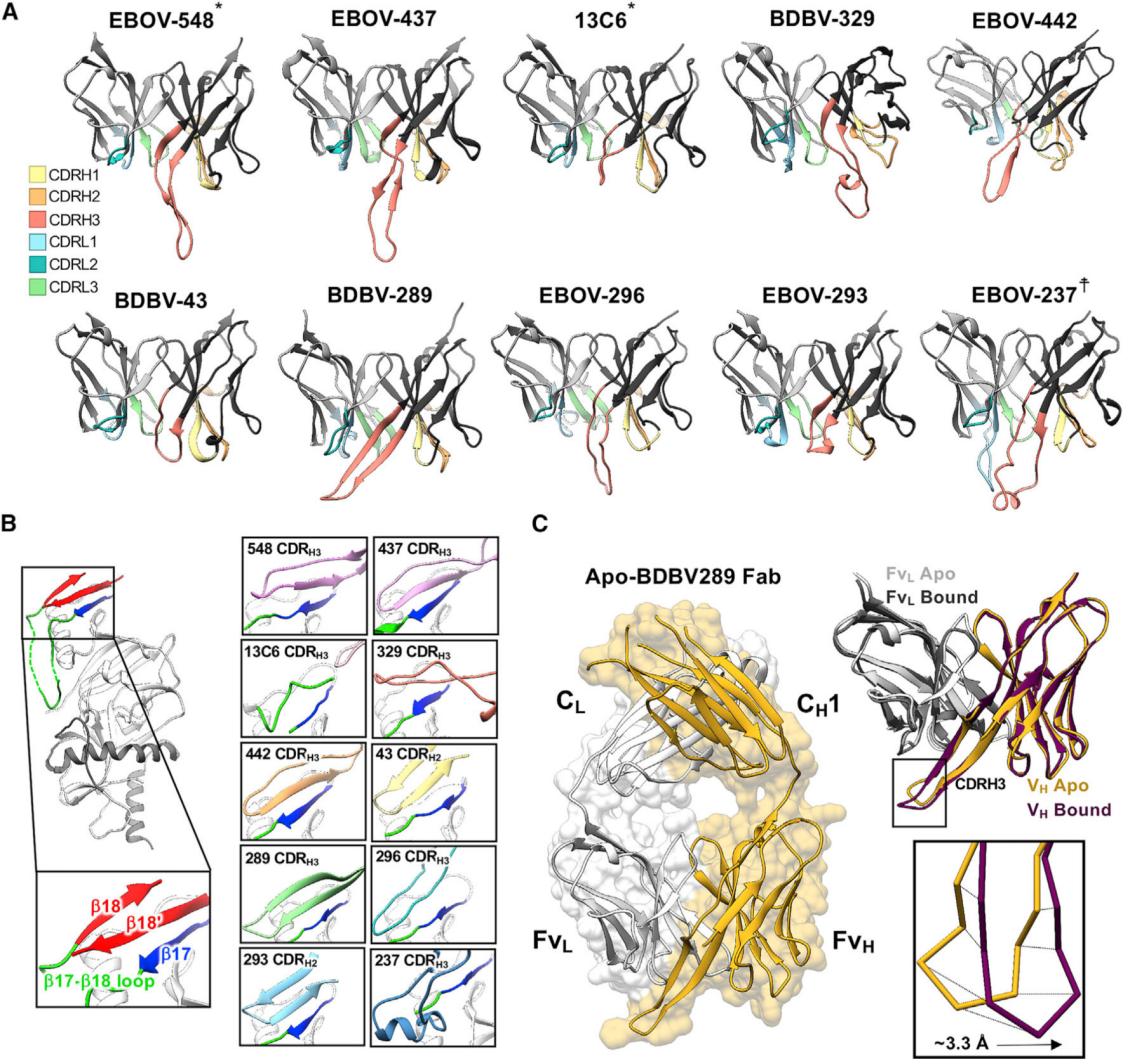
Glycan cap antibody paratopes feature CDR loops with β-hairpin structures that mimic and displace the β18-β18′ region in the glycan cap (A) Ribbon models of the glycan cap antibody Fv domains with CDR loops highlighted. The HC is in dark gray (right) and the LC is in light gray (left). (B) Structures highlighting the interaction of each of the glycan cap antibodies with the β17 strand, which forms the basis of an extended b sheet in the glycan cap with the β17-β18 loop and β18-β18′ hairpin motif (shown on the left). (C) Crystal structure of the BDBV-289 Fab. Shown on the right is a comparison of the apo- and GP-bound forms of BDBV-289. *, from a previous study; †, shown as an initial homology model. EBOV-548 (PDB: 6UYE) and 13C6 (PDB: 5KEL) are included for comparison. See also Figure S5 and Tables S3 and S5.

The CDRH3 loops of the glycan cap antibodies generally adopt an extended β-hairpin motif with a partial or full β strand secondary structure (Figure 4A). These loops also pair with β17 in GP1 to form an extended β sheet and displace the β18-β18′ hairpin by mimicking its structure, as observed in our previous structure of EBOV-548 (Figure 4B). Alternatively, BDBV-43 and EBOV-293 use shorter CDRH2 loops to pair with β17 (Figures 4A and 4B). Conversely, 13C6 has a much shorter CDRH3 loop and does not make full contact with β17 (Figure 4B), possibly explaining its lack of synergy with base antibodies (Figure 1A). EBOV-237 and BDBV-329 are unique among the antibodies we examined here because of very long CDRH3 loops at 25 or 33 amino acids, respectively.

We also determined the unliganded crystal structure of BDBV-289 Fab to 3.0-Å resolution to compare the conformations of the CDR loops prior to GP engagement (Figure 4C; Table S5). The structure of the unliganded BDBV-289 Fab is very similar to BDBV-289 Fab bound to EBOV GPΔMuc, with a root-mean-square deviation (RMSD) of 1.6 Å for the Fv portions of the HC and light chain (LC) (Figure 4C). There is a slight shift of the CDRL3 to accommodate the α2-β17 loop in the glycan cap and a larger shift of CDRH3 (Figure 4C). In the GP-bound structure, the CDRH3 loop moves toward GP by an average distance of ~3.3 Å (Figure 4C). In the crystal structure, this movement is blocked by a crystal lattice interaction, but this difference may indicate flexibility in the tip of this loop.

### The β18-β18′ hairpin anchors the MLDs and shields a conserved hydrophobic patch in the glycan cap

**Figure 5.**
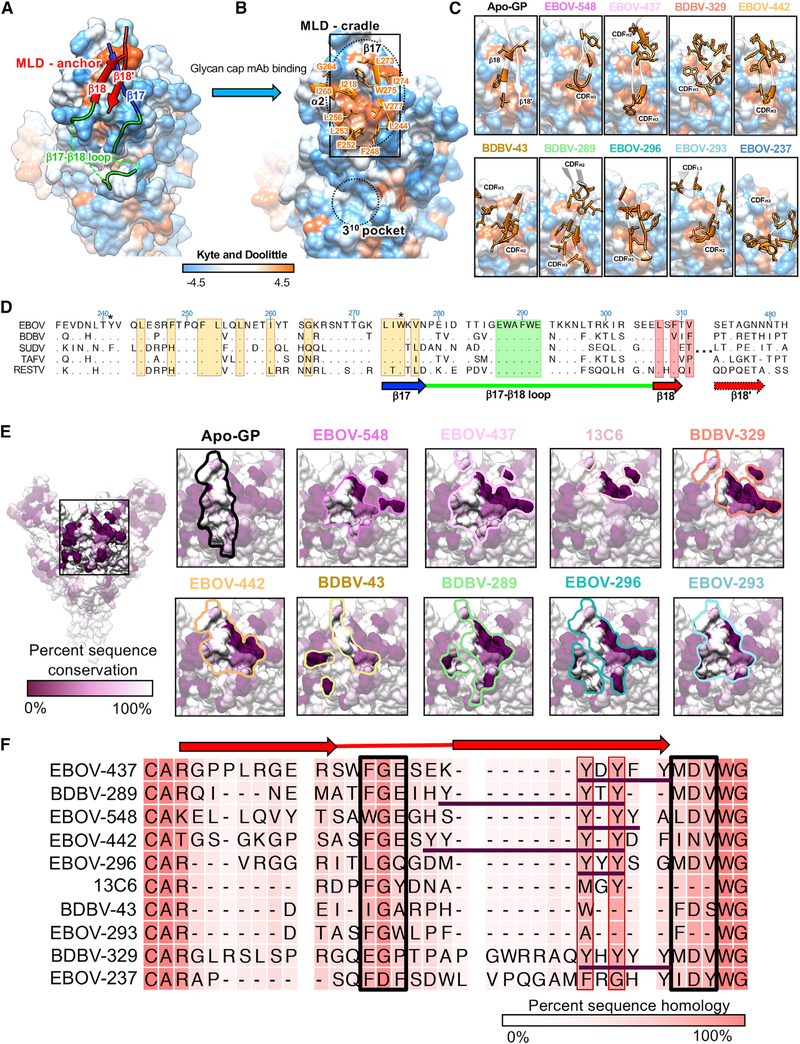
Glycan cap antibodies target a conserved hydrophobic cradle that anchors the MLDs to GP1 (A) Hydrophobicity surface rendering of the apo-EBOV GP protomer (PDB: 5JQ3), with the MLD anchor (β18-β18′) highlighted in red. Using the Kyte and Doolittle scale (Kyte and Doolittle, 1982), hydrophobic residues are colored orange and hydrophilic ones in blue. (B) Upon glycan cap mAb binding, the MLD anchor is displaced, exposing a hydrophobic pocket we call the MLD cradle. The cradle lies within a groove formed by α2 and β17, directly above the 3^10^ pocket. Key residues of the cradle are indicated. The MLD cradle is composed of residues from α2 and β17 as well as some additional residues that lie deeper in the core of GP1, including I218, F248, F252, L253, L256, I260, G264, L273, I274, W275, V277, and L244. The cradle is segmented in the middle by W275, which may explain this residue’s pivotal role in binding of many glycan cap antibodies to GP. (C) Interaction of glycan cap mAb HC loops with the MLD cradle (from the rectangle in B). Key hydrophobic residues from antibody paratopes are indicated. (D) Sequence alignment of the MLD anchor and cradle epitope for the five main ebolaviruses (EBOV; GenBank: QQN67572.1; BDBV, GenBank: AYI50382.1;SUDV, GenBank: AGB56678.1; Tai Forest virus [TAFV], GenBank: AWK96625.1; and Reston virus [RESTV], GenBank: QNF60335.1), with topology indicated below. Residues highlighted in orange are key hydrophobic residues that form the cradle; those in green form the base of the β17-β18 loop that interact with the base of the fusion loop, and those in red are key residues from β18 that interact with the cradle in apo-GP. Those marked with an asterisk are common escape mutants for this epitope. (E) Glycan cap antibody footprints highlighted on the structure of apo-GP, colored to reflect conservation, with dark purple indicating complete lack of conservation and white indicating complete conservation. (F) Sequence alignment of the CDRH3 region from each of the glycan cap antibodies analyzed in this study, with darker pink indicating complete conservation andlight pink indicating complete lack of conservation. The beta-turn-beta structure common to these paratopes is indicated above. Key sequences that are similar among these antibodies are boxed in black, with “Y” stretches from *IGHJ6* gene use underlined in purple. See also Figure S5.

The β18-β18′ region of the glycan cap forms a β-hairpin that anchors the MLD, forming an extended β sheet with the underlying core of GP1 (Figure 5A; Zhao et al., 2016). Because of the recurrence of the β18-β18′ hairpin epitope within the glycan cap and its role in anchoring MLD, we have called this portion of the glycan cap the MLD anchor (Figure 5A). Upon binding of the glycan cap antibody, the MLD anchor is displaced, revealing a patch of hydrophobic residues, which we refer to as the MLD cradle (Figure 5B).

The MLD anchor contains complementary hydrophobic residues along the β18′ strand that are buried by the MLD cradle (Figure 5B). Through molecular mimicry, the CDRH3 or CDRH2 loops of each of the neutralizing glycan cap antibodies characterized in this study bury analogous hydrophobic residues in the cradle, displacing the anchor (Figure 5C). Our structures of EBOV-548 (Gilchuk et al., 2020) Fab and BDBV-289 Fab bound to GP indicate that, although binding abrogates attachment of the MLD anchor, the β17-β18 loop most likely remains tethered to the base of the IFL via W291_GP1_ to N512_GP2_. However, glycan cap binding may remove some restraint on the β17-β18 loop, allowing increased binding by GP base-directed antibodies.

Notably, we observed that the MLD cradle forms a hydrophobic pocket on the outside of the GP, which is accessed by side chains of the glycan cap antibody CDRH2 or CDRH3 loops (Figure 5C). The extent to which contacts are made within this pocket correlates positively with antibody-induced trimer instability as well as synergy with base antibodies (Figure 1). For example, EBOV-548, EBOV-437, and EBOV-442 utilize W108_H3_, F109_H3_, and F113_H3_, respectively, to fully fill this pocket (Figures 3F, 3I, and 5C). Together with EBOV-237, which also contains similar residues that likely bind this region based on our low-resolution model, these antibodies exhibit the greatest level of trimer instability and synergy (Figure 1). The antibody EBOV-296 uses M112_H3_ to access this pocket and produces an intermediate level of trimer instability and synergy (Figures 1 and 3J). Conversely, EBOV-293, EBOV-289, BDBV-329, and BDBV-43 only partially contact this pocket and also exhibit lower or no trimer instability (Figure 1).

The sequence of the N-terminal portion of the MLD anchor (β18), the MLD cradle, and the β17-β18 loops are relatively conserved throughout all EBOVs but the surrounding regions in the glycan cap are not (Figure 5D). The glycan cap antibody contacts described here are focused on the conserved β17 epitope and the key residue at W275, but additional contacts outside of this region are also observed (Figure S4; Table S4).

The footprints of the antibody epitopes on GP vary in terms of the extent and level of inclusion of less conserved residues (Figure 5E; Figure S4). In addition to differences in the size and shape of glycan cap antibody epitope footprints, the number of contacts associated with residues also varies for each antibody, with some antibodies relying more heavily on less conserved GP residues, such as BDBV-329, and others having very few non-conserved contacts, such as EBOV-293 (Figure S4).

### Germline analysis and conservation of features within glycan cap antibody paratopes

The glycan cap antibodies described here share several common features, including the majority (5 of 9) deriving from the *IGHV-169* germline gene segment (Table S2). The Immunogenetics database (http://www.imgt.org) currently lists 19 *IGHV1–69* human alleles that are often distinguished by a phenylalanine (F) or leucine (L) polymorphism at position 54 (Kabat numbering) critical for some broadly neutralizing antibodies (Lingwood et al., 2012). Frequent use of the *IGHV1–69* gene is common in the antibody repertoires of those infected by influenza virus (Lang et al., 2017), HCV (Chan et al., 2001), HIV-1 (Huang et al., 2004), and other pathogens (Chen et al., 2019). The *IGHV1–69* gene is thought to be superior for viral neutralization at certain epitopes because of the presence of key germline-encoded hydrophobic residues, especially in CDRH2, as well as for breadth because of a large repertoire of allelic and copy number variations (Chen et al., 2019). Despite a wide range of donors, we also found these characteristics to be present in the EBOV antibodies described here (Table S2; Figure S5).

Use of *IGHV1–69* imparts a germline-encoded CDRH2 with several hydrophobic residues that is used by BDBV-43 and EBOV-293 to bind to the MLD cradle (Figure 5C). Coincidentally, BDBV-43 and EBOV-293 use the *IGHV1–69* L polymorphism, but this does not appear to be a critical factor in their binding. BDBV-43 and EBOV-293 also have much shorter CDRH3 loops, which may be a consequence of their use of CDRH2 to bind the MLD cradle (Figure S5). All of the glycan cap antibodies analyzed here use a combination of heavy-chain genes with germline-encoded hydrophobic residues on either side of the CDRH3 gene (Figure S5). This feature may potentiate the stability of the longer CDRH3 loops we observed here. Overall, somatic hypermutation (SHM) was generally high throughout all glycan cap mAbs studied here, with an average of ~11% or ~6% amino change from germline for the V_H_ or V_L_ regions, respectively (Table S2).

Despite varying CDRH3 length, the tip of the CDRH3 hairpin contains a highly conserved glycine surrounded by hydrophobic residues and a C-terminal tyrosine motif (Figure 5F). This glycine and the hydrophobic tip help to insert the CDRH3 loop into the MLD cradle (Figures 5A–5C) and assists with formation of the hairpin structure necessary for proper binding. The C-terminal tyrosine motif stabilizes longer CDRH3 loops within the core of the paratope and provides additional, non-specific hydrophobic contacts within the core of the epitope.

**Figure 6.**
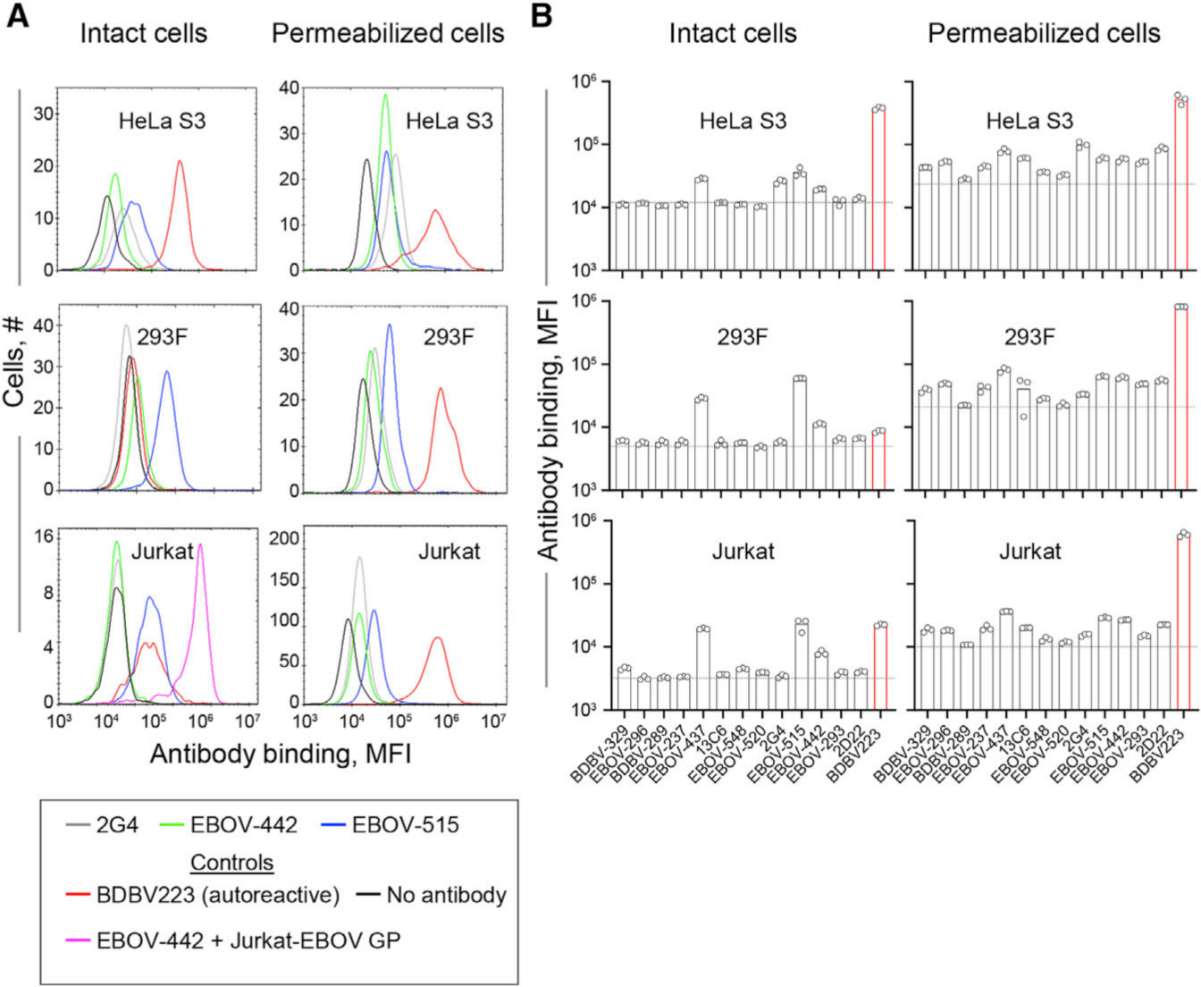
Antibody reactivity to the human HeLa S3, 293F, and Jurkat cell lines Intact or fixed and permeabilized cells were stained with 5 μg/mL of individual mAbs, followed by incubation with the detection phycoerythrin (PE)-conjugated anti-human immunoglobulin G (IgG) antibody and flow cytometry analysis. (A) Representative flow cytometry histograms showing binding of broadly neutralizing mAbs EBOV-442 (green) and EBOV-515 (blue) and the monoreactive mAb 2G4 (gray) to the indicated human cell lines. Binding of EBOV-442 to Jurkat-EBOV GP cells served as a control for antigen-specific binding (magenta). The mAb BDBV223 with known autoreactivity served as a control for autoreactivity (red). Cells stained with the detection PE-conjugated anti-human IgG antibody served as a control for the assay background. Cells were identified based on forward and side scatter analysis. (B) Median fluorescence intensity (MFI) quantifying binding to intact (extracellular staining) or fixed and permeabilized (extracellular and intracellular staining) cells of each antibody tested. The data are shown as a scatterplot of individual values from triplicate measurements for each mAb, with bar indicating the mean. Dotted line indicates the background level from the detection of antibody binding only as described in (A).

Broadly neutralizing activity of anti-viral human antibodies is often associated with poly- or autoreactivity (Bajic et al., 2019; Liu et al., 2015). To examine the autoreactivity of the panel of the EBOV GP-directed mAbs, we used suspension-grown HeLa S3, 293F, and Jurkat human cell lines and a quantitative high-throughput flow cytometry assay for antibody autoreactivity assessment that we described previously (Mousa et al., 2017). This analysis revealed that GP-specific, broadly reactive mAbs exhibit a low to intermediate level of autoreactivity compared with a known IGHV4–34*01-encoded autoreactive EBOV GP membrane proximal external region (MPER)/heptat repeat 2 (HR2)-specific mAb designated BDBV223 (Flyak et al., 2018; King et al., 2019; Sabouri et al., 2014) or to an antigen-specific mAb control (Figure 6). Therefore, we concluded that *IGHV1–69* gene use was not associated with higher autoreactivity for the panel of mAbs tested.

### Glycan cap antibodies inhibit cleavage

**Figure 7.**
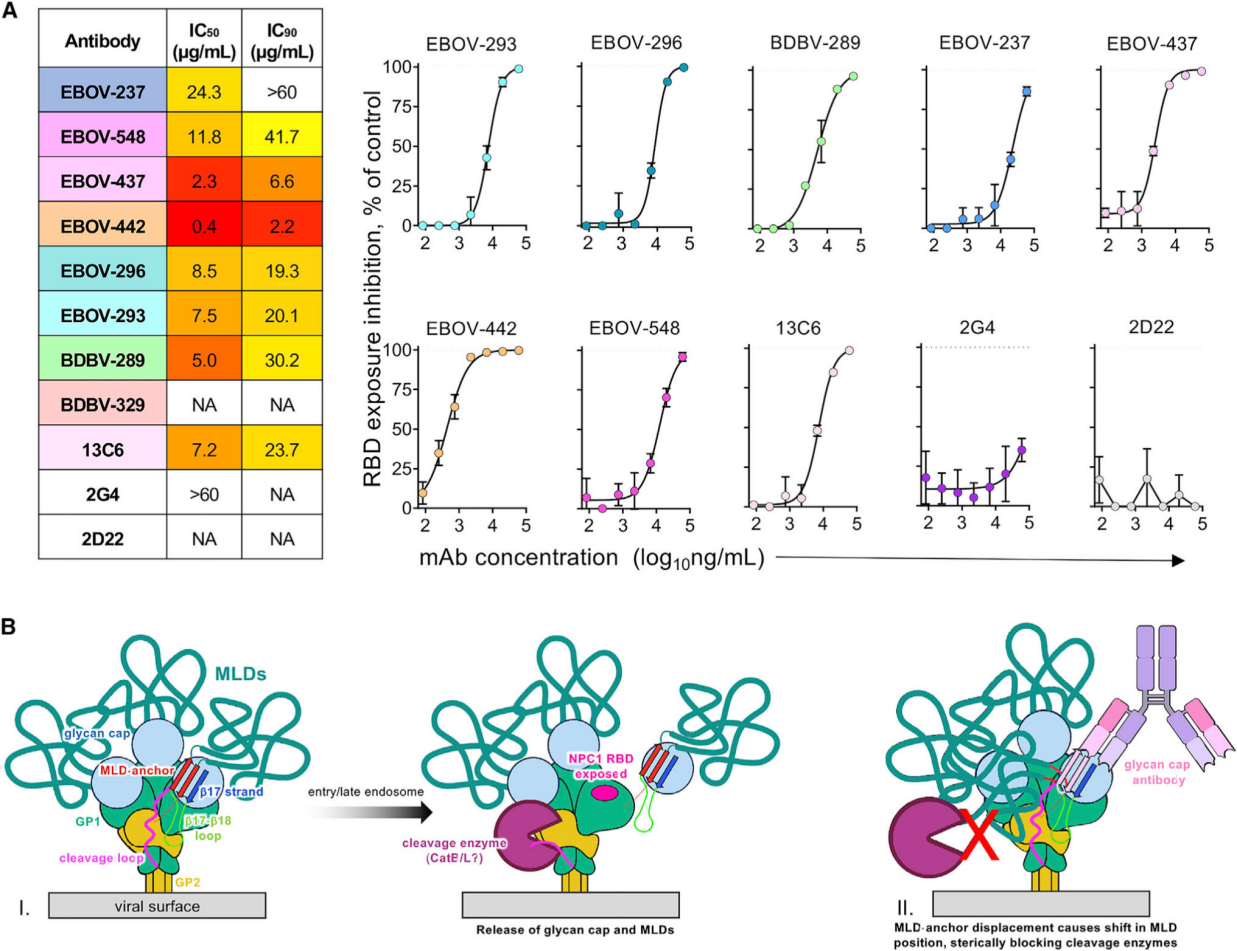
Cleavage inhibition by glycan cap antibodies (A) The Jurkat-EBOV GP was incubated with various concentrations of antibodies, treated with thermolysin, and then assayed using flow cytometry for exposure of the receptor binding site (RBS), as measured by binding of a fluorescently labeled MR78 antibody that recognizes the RBS. 50% inhibitory concentration (IC_50_) and 90% inhibitory concentration (IC_90_) values (left) and dose-dependent inhibition curves (right) are shown. Dotted line indicates percentage of RBS exposure in the presence of the 2D22 control. BDBV-329 was excluded because it does not bind to the EBOV GP, and BDBV-43 was not tested because of poor recombinant expression. Mean ± SD of technical triplicates from one experiment is shown. (B) Proposed model of GP inhibition by glycan cap antibodies (I) Enzyme cleavage of a loop draped over the outside of the GP (magenta) is thought to release the glycan cap and attached MLD. (II) Glycan cap antibodies that bind to the MLD cradle displace the MLD anchor and, thus, the MLDs themselves, potentially shifting their position and sterically blocking access to the cleavage loop by enzymes, especially on a GP-dense viral surface. See also Figure S6.

The underlying molecular mechanism for how an antibody neutralizes is related to its ability to inhibit viral infection, which can be achieved by diverse mechanisms, including cleavage inhibition. To determine the ability of the antibodies used in this study to inhibit cleavage, we performed a cleavage-blocking assay, as described previously (Gilchuk et al., 2018; Figure 7A). Jurkat cells stably transduced with EBOV GP (Jurkat-EBOV GP) were pre-incubated with individual antibodies, followed by treatment with thermolysin to mimic cathepsin cleavage to yield membrane-displayed GP_CL_ (Jurkat-EBOV GP_CL_). Exposure of the RBS on GP_CL_ was measured by the level of binding of the fluorescently labeled RBS-specific mAb MR78, which does not bind uncleaved EBOV GP (Flyak et al., 2015) The epitope of glycan cap antibodies is being removed by cleavage, and in a separate assay, we confirmed that none of tested antibodies, except EBOV-442, compete with MR78 on Jurkat-EBOV GP_CL_ (Figure S6). EBOV-442 partially competed with MR78 (Figure S6), suggesting incomplete removal of its epitope by thermolysin, which may have a minor effect on quantification of cleavage inhibition by this antibody. All EBOV GP-reactive glycan cap antibodies revealed dose-dependent cleavage inhibition, and most of them fully blocked cleavage at the highest tested concentration of 60 μg/mL (Figure 7A). The base antibody 2G4 and the control antibody 2D22 did not inhibit cleavage. Although the glycan cap antibodies in this study do not interact directly with the cathepsin cleavage loop, disruption or dislocation of the MLD may be an obstacle for recognition or cleavage activity by enzymes (Figure 7B).

## DISCUSSION

Our previous structure of the EBOV-548/EBOV GP complex first revealed the glycan cap binding site containing the β18-β18′ hairpin (Gilchuk et al., 2020); however, the extensive structural evidence we provide here more completely describes this epitope, which we call the MLD anchor and cradle. Displacement of the MLD anchor suggests that it is bound transiently, similar to the β17-β18 loop (West et al., 2019). Anecdotally, we and others have often noticed that the glycan cap is not well resolved in negative stain and cryo-EM structures of GPs that lack coordinating glycan cap antibodies, suggesting that this entire domain may be loosely attached to the GP. This transient structural feature may aid with removal of the glycan cap upon cleavage for exposure of the NPC1 binding site. The MLD anchor makes very limited contact with the underlying hydrophobic cradle, essentially mediated by a single β strand. These characteristics have been observed for other antibodies that bind with hydrophobic hairpin CDR loops, suggesting a conserved mechanism for neutralization that extends to other viruses (Lee et al., 2017; Pancera et al., 2010; Yuan et al., 2019).

Despite a high level of overlap in the glycan cap epitope for the antibodies studied here, there are significant differences in their degree of pan-EBOV binding and neutralization (Table S1). For example, BDB-329 and EBOV-237 only bind and neutralize BDBV and EBOV, respectively, whereas EBOV-293 and EBOV-442, for example, bind and neutralize viruses outside of their autologous virus (Table S1). Notably, however, some cross-reactive antibodies only potently neutralize their autologous virus, such as EBOV-437 and, to a lesser extent, BDBV-43 and BDBV-298 (Table S1). Our structures provide the molecular basis for reactivity breadth and suggest why cross-reactive glycan cap antibodies are rare in the immune repertoire of survivors. BDBV-329 and EBOV-237 make extensive contacts across the GP, including several non-conserved residues, which likely render them single species specific. However, BDBV-293 and EBOV-442 have much smaller footprints (Figure 5E). Therefore, the most broadly neutralizing glycan cap antibodies generally limit their engagement of GP to just the MLD cradle and make minimal contacts in regions outside of this epitope that are less conserved.

Several antibodies in this study also use the *IGHV1–69* gene and long CDRH3 loops to access β17 and the MLD cradle epitope. The MLD cradle itself is very hydrophobic (Figure 5B) and, therefore, may be well suited for engaging germline genes with complementary features such as *IGHV1–69*. However, it is possible that contact with CDRH3 loops by germline-encoded, bulky hydrophobic residues (Figure S5) rather than strict use of *IGHV1–69* is the key feature in selection of germline genes for this class of neutralizing antibodies. BDBV-43 and EBOV-293 have CDRH3s that share this feature but bind to GPs with geometries that restrict access of the MLD anchor by their CDRH3. Therefore, BDBV-43 and EBOV-293 alternatively utilize their CDRH2 to access this epitope, which subsequently undergoes a large degree of SHM. Comparatively, antibodies that do not use their CDRH2 to access the MLD anchor have much lower SHM in this loop. For antibodies with geometries allowing CDRH3 access to the MLD anchor, CDRH3s with above-average length are observed. Our structures indicate that such long CDRH3s are necessary to fully access the MLD cradle in these cases. Our observations suggest that use of longer CDRH3s may destabilize the trimer and aid with cooperative binding of base antibodies (Figure 1).

The glycan cap antibodies described here generally have high levels of SHM, with EBOV-293 containing 24 mutations from the inferred germline gene in its heavy chain (Table S2). This count also does not consider potential somatic mutations in the long CDRH3 loops, whose germline origins cannot be predicted but likely arise from large numbers of N additions during the original V-D-J recombination event. How glycan cap antibody SHM compares to mutation frequency in antibodies directed toward other epitopes is not well explored, but the amount of SHM we observe for these neutralizing glycan cap mAbs is higher than what is generally reported in EBOV survivor repertoires (Davis et al., 2019). Glycan cap antibodies are now known to form a large portion of the adaptive response to natural infection (Bornholdt et al., 2016b; Flyak et al., 2016; Wec et al., 2017). Several of these antibodies can potently neutralize; however, they are often mono-specific. It is unclear how the smaller subset of rarer, broadly neutralizing glycan cap antibodies develops. Our observations indicate that they may require higher levels of SHM combined with structural adaptations to reach cryptic epitopes shielded by the MLD, the MLD anchor, and glycans.

The mechanism of viral neutralization by glycan cap antibodies is unclear. These antibodies could potentially act indirectly by preventing access to a cleavage loop that is necessary to cleave during viral entry (Bornholdt et al., 2016a; Figure 7B, part I). The MLDs are large, accounting for over half of the mass of GPs, and are unstructured and highly glycosylated. Although the MLDs on EBOVs are known to sit atop the GP, those on marburgviruses are thought to drape over the sides (Hashiguchi et al., 2015). This difference may occur because marburgviruses lack the structured glycan cap that is found in EBOVs (King et al., 2018). Consequently, the NPC1 RBS is fully exposed on full-length GP in marburgviruses (Flyak et al., 2015), whereas it is hidden under the glycan cap and MLD on EBOVs. Therefore, the MLD anchor appears to pin the MLD down to the top of EBOV GPs, keeping them above the GP and out of the way of the cleavage loop. Displacing the MLD anchor may displace the MLDs themselves while retaining covalent attachment of these large domains to the GP (Figure 7B, part II). In the dense environment of the EBOV surface, in which many GP spikes are known to crowd together in close proximity (Tran et al., 2014), this displacement may cause the MLD to drape over the cathepsin cleavage loops, blocking access by enzymes.

Overall, our data collectively provide the molecular basis for synergy, breadth of reactivity, and virus neutralization by potent glycan cap-directed antibodies and suggest a rational strategy for design of broad therapeutic antibody cocktails.

## STAR★METHODS

### RESOURCE AVAILABILITY

#### Lead contact

Further information regarding requests for resources and reagents should be directed to and will be fulfilled by the Lead Contact, Andrew Ward (andrew@scripps.edu).

#### Materials availability

Plasmids generated in this study are available upon request by the Lead Contact.

#### Data and code availability

The cryo-EM maps and structural coordinates generated during this study are available at the Electron Microscopy Data Bank (https://www.ebi.ac.uk/pdbe/emdb) and the Worldwide Protein Data Bank (https://www.pdb.org). The accession codes for the following cryo-EM maps reported in this paper are: EMD-22839 (EBOV GPΔMuc:BDBV289 Fab), EMD-22841 (BDBV GPΔMuc:BDBV43 Fab and ADI-15878 Fab), EMD-22853 (EBOV GPΔMuc:EBOV-437 Fab and EBOV-515 Fab), EMD-22848 (EBOV GPΔMuc:EBOV-442 Fab and EBOV-515 Fab), EMD-22842 (EBOV GPΔMuc:EBOV-293 Fab and EBOV-515 Fab), EMD-22847 (EBOV GPΔMuc:EBOV-296 Fab and EBOV-515 Fab), EMD-22851 (EBOV GPΔMuc:BDBV-329 Fab and EBOV-515 Fab) and EMD-22852 (EBOV GPΔMuc:EBOV-237 Fab and EBOV-515 Fab). The accession codes for PDB files are: 7KEJ (EBOV GPΔMuc:BDBV-289 Fab), 7KEW (BDBV GPΔMuc:BDBV-43 Fab), 7KFH (EBOV GPΔMuc:EBOV-437 Fab), 7KFB (EBOV GPΔMuc:EBOV-442 Fab), 7KEX (EBOV GPΔMuc:EBOV-293 Fab), 7KF9 (EBOV GPΔMuc:EBOV-296 Fab), 7KFE (EBOV GPΔMuc:BDBV-329 Fab) and 7KFG (unliganded BDBV289 Fab).

### EXPERIMENTAL MODEL AND SUBJECT DETAILS

#### Human samples

Human PBMCs were obtained from a survivor of the 2014 EVD epidemic who acquired the infection in the Democratic Republic of Congo and was treated in the Nebraska Medical Center in the United States. A male human survivor was age 57 when PBMCs were collected. PBMCs were collected after the illness had resolved, following written informed consent. The studies were approved by the Institutional Review Board of Vanderbilt University Medical Center.

#### Cell lines

Suspension adapted HEK293F cells were obtained from ThermoFisher Scientific and cultured in serum-free FreeStyle medium. Cells were maintained in shaking incubators at 100% humidity, 37°C and 8% CO_2_. Expi293F cells (ThermoFisher Scientific) were maintained at 37 °C in 8% CO_2_ in Expi293F Expression Medium (ThermoFisher Scientific). ExpiCHO cells (ThermoFisher Scientific) were maintained at 37°C in 8% CO_2_ in ExpiCHO Expression Medium (ThermoFisher Scientific). The Jurkat-EBOV GP (variant Makona) cell line stably transduced to display respective GP on the surface (Davis et al., 2019) was a kind gift from Carl Davis (Emory University, Atlanta, GA). Jurkat-EBOV GP cells were maintained at 37°C in 8% CO_2_ in RPMI-1640 medium (GIBCO) supplemented with 10% fetal heat-inactivated fetal bovine serum (FBS). Mycoplasma testing of Expi293F and ExpiCHO cultures was performed on a monthly basis using a PCR-based mycoplasma detection kit (ATCC). Cell lines were not authenticated following purchase.

#### Viruses

Mouse-adapted EBOV Mayinga (EBOV-MA, GenBank: AF49101) virus was described previously (Bray et al., 1998).

#### Mouse models

Seven- to eight-week old female BALB/c mice were obtained from the Jackson Laboratory. Mice were housed in microisolator cages and provided food and water *ad libitum*. Challenge studies were conducted under maximum containment in an animal biosafety level 4 (ABSL-4) facility of the Galveston National Laboratory, UTMB. The animal protocols for testing of mAbs in mice were approved by the Institutional Animal Care and Use Committee (IACUC) of the University of Texas Medical Branch in compliance with the Animal Welfare Act and other applicable federal statutes and regulations relating to animals and experiments involving animals.

### METHOD DETAILS

#### Isolation of mAbs EBOV-293 and EBOV-296

Hybridoma cell lines secreting human mAbs were generated as described previously (Flyak et al., 2018). In brief, previously cryopreserved PBMC samples were transformed with Epstein-Barr virus, CpG and additional supplements. After 7 days, cells from each well of the 384-well culture plates were expanded into four 96-well culture plates using cell culture medium containing irradiated heterologous human PBMCs (recovered from blood unit leukofiltration filters, Nashville Red Cross) and incubated for an additional four days. Plates were screened for EBOV GP antigen-specific antibody-secreting cell lines using enzyme-linked immunosorbent assays (ELISAs). Cells from wells with supernatants reacting with antigen in an ELISA were fused with HMMA2.5 myeloma cells using an established electrofusion technique (Yu et al., 2008). Antibody heavy- and light-chain variable region genes were sequenced from hybridoma lines that had been cloned biologically by single-cell flow cytometric sorting. Briefly, total RNA was extracted using the RNeasy Mini kit (QIAGEN) and reverse-transcriptase PCR (RT-PCR) amplification of the antibody gene cDNAs was performed using the PrimeScript One Step RT-PCR kit (CLONTECH) according to the manufacturer’s protocols with gene-specific primers (Thornburg et al., 2016). The thermal cycling conditions were as follows: 50°C for 30 min, 94°C for 2 min, 40 cycles of (94°C for 30 s, 58°C for 30 s and 72°C for 1 min). PCR products were purified using Agencourt AMPure XP magnetic beads (Beckman Coulter) and sequenced directly using an ABI3700 automated DNA sequencer. The identities of gene segments and mutations from germlines were determined by alignment using the ImMunoGeneTics database (Giudicelli and Lefranc, 2011).

#### Synergistic binding to cell-surface-displayed GP

The assay was performed as described previously (Gilchuk et al., 2018). Briefly, Jurkat-EBOV GP cells were pre-incubated at 4°C for 30 min with individual unlabeled glycan cap-specific mAbs at a saturating for GP binding concentration (20 μg/mL) in PBS containing 2% FBS and 2 mM EDTA, and then Alexa Fluor 647-labeled mAbs EBOV-515 or EBOV-520 were added to a total concentration of labeled mAbs of 10 μg/mL. Cells were incubated at 4°C for additional 2 h, then washed and antibody binding was analyzed by flow cytometry using an iQue Screener Plus flow cytometer (Intellicyt). Controls included binding of labeled mAb to mock-transduced Jurkat cells (background), binding of labeled mAb alone to intact Jurkat-EBOV GP (a baseline level of binding to calculate fold change in a presence of glycan mAb), and binding of labeled mAb alone to cleaved Jurkat-EBOV-GP (maximal saturating binding signal). Results are expressed as fold-increase in median fluorescence intensity (MFI) of labeled mAb binding in the presence of the tested unlabeled mAb minus background signal from mock control.

#### ELISA binding assays

To assess mAb binding, wells of 96-well microtiter plates were coated with purified, recombinant EBOV, BDBV or SUDV GPΔTM ectodomains or EBOV sGP at 4°C overnight. Plates were blocked with 2% non-fat dry milk and 2% normal goat serum in DPBS containing 0.05% Tween-20 (DPBS-T) for 1 h. Purified mAbs were diluted serially in blocking buffer, added to the wells and incubated for 1 h at ambient temperature. The bound antibodies were detected using goat anti-human IgG conjugated with horseradish peroxidase (Southern Biotech) diluted in blocking buffer and TMB substrate (ThermoFisher Scientific). Color development was monitored, 1N hydrochloric acid was added to stop the reaction, and the absorbance was measured at 450 nm using a spectrophotometer (Biotek).

#### Epitope mapping using an EBOV GP alanine-scan mutation library

Epitope mapping was carried out as described previously (Davidson et al., 2015). Comprehensive alanine scanning (‘shotgun mutagenesis’) was carried out on an expression construct for EBOV GP (Yambuku-Mayinga variant) lacking the mucin-like domain (residues 311–461), mutagenizing GP residues 33–310 and 462–676 to create a library of clones, each representing an individual point mutant. Residues were changed to alanine (with alanine residues changed to serine). The resulting library, covering 492 of 493 (99.9%) of target residues, was arrayed into 384-well plates, one mutant per well, then transfected into HEK293T cells and allowed to express for 22 hr. Cells, unfixed or fixed in 4% paraformaldehyde, were incubated with primary antibody and then with an Alexa Fluor 488-conjugated secondary antibody (Jackson ImmunoResearch Laboratories). After washing, cellular fluorescence was detected using the Intellicyt flow cytometer. mAb reactivity against each mutant EBOV GP clone was calculated relative to wild-type EBOV GP reactivity by subtracting the signal from mock-transfected controls and normalizing to the signal from wild-type GP-transfected controls. Mutated residues within clones were identified as critical to the mAb epitope if they did not support reactivity of the test mAb but did support reactivity of other control EBOV mAbs. This counter-screen strategy facilitated the exclusion of GP mutants that were misfolded locally or that exhibited an expression defect. The detailed algorithms used to interpret shotgun mutagenesis data were described previously (Davidson and Doranz, 2014).

#### Mouse challenge and protection studies

Groups of 7–8-week-old female BALB/c mice (n = 5 per group) housed in microisolator cages were inoculated with 1,000 PFU of the EBOV-MA by the intraperitoneal (i.p.) route. Mice were treated i.p. with 100 μg (~5 mg/kg) of individual mAb per mouse on 1 day post inoculation (dpi). Human mAb 2D22 (specific to an unrelated target, dengue virus) served as a negative control (Fibriansah et al., 2015). Mice were monitored twice daily from day 0 to 14 post infection for illness, survival, and weight loss, followed by once daily monitoring from 15 dpi to the end of the study at 28 dpi. The extent of disease was scored using the following parameters: dyspnea (possible scores 0–5), recumbence (0–5), unresponsiveness (0–5), and bleeding/hemorrhage (0–5). Moribund mice were euthanized as per the IACUC-approved protocol. All mice were euthanized on day 28 after EBOV challenge.

#### Cryo-EM trimer stability assay

Complexes for trimer stability assays were derived from data collected for structural evaluation (*see* Cryo-EM sample preparation section below). Particle picks were completed using a difference of Gaussian method with low thresholds in order to pick everything on the grids. Particles were separated into stacks for either intact particles or particles that were falling apart, which was judged by eye, and then counted to determine approximate percentage of glycan cap antibody-induced instability. We have previously determined that base binding antibodies alone do not induce trimer instability.

#### Assessing autoreactivity of mAbs by flow cytometry

Cultures of Jurkat E6–1 (ATCC) and Jurkat-EBOV GP cells were grown in RPMI-1640 medium supplemented with 10% fetal bovine serum (FBS, HyClone) according to the ATCC recommendations. FreeStyle 293F cells (ThermoFisher Scientific) were cultured in serum-free FreeStyle medium in shaking incubator, at 37°C and 8% CO_2_. Suspension-adapted HeLa S3 cells (ATCC) were cultured in F-12 medium supplemented with 10% FBS (HyClone) at 37°C and 5% CO_2_ without shaking.

To analyze mAb binding to extracellular proteins, cells were washed with ice-cold FACS buffer (Dulbecco’s PBS containing 2% FBS and 2 mM EDTA), counted, seeded at ~5,000 to 20,000 viable cells per well in a V-bottom 96-well plate for each mAb to be tested and incubated 2 h at 4°C with 5 μg/mL of mAb in triplicate in a total volume 50 μL per staining. Cells were washed twice with FACS buffer by centrifugation for 2 min at 450 × *g* followed by incubation with a 1:500 dilution of the detection phycoerythrin (PE)-conjugated anti-human IgG Fc antibody (multi-species IgG pre-absorbed; Southern Biotech) in FACS buffer. To analyze mAb binding to intracellular proteins, cells were washed with ice-cold Dulbecco’s PBS and fixed with Cytofix/Cytoperm Fixation and Permeabilization Solution (BD Biosciences). Fixed cells were washed twice with 1 × BD Perm/Wash Buffer and incubated with the PE-conjugated anti-human IgG antibody in 1 × BD Perm/Wash Buffer. After washing of stained cells, 200 to 1,000 cell events were acquired using an iQue Screener Plus flow cytometer (Intellicyt). Cells were identified based on forward and side scatter analysis, and a median fluorescence intensity of PE staining was determined using ForeCyt software (Intellicyt).

#### Cell surface displayed GP mAb competition-binding

Jurkat-EBOV GP_CL_ cells were pre-incubated with a saturating concentration (typically 20 μg/mL) of glycan cap mAbs at room temperature for 30 min, followed by addition of labeled antibody MR78 (Flyak et al., 2015; Hashiguchi et al., 2015) at 5 μg/mL and incubated for an additional 30 min. Antibody MR78 was labeled with Alexa Fluor 647 and added after the first mAb and without washing of cells to minimize a dissociation of the first mAb from cell surface GP during a prolonged incubation. Cells were washed, fixed with 4% paraformaldehyde, and cell staining was analyzed using an iQue Screener Plus flow cytometer flow cytometer. Background values were determined from binding of the second labeled mAbs to untransfected Jurkat. Results are expressed as the percent of binding in the presence of glycan cap mAb relative to MR78 alone (maximal binding) minus background. The antibodies were considered competing if the presence of first antibody reduced the signal of the second antibody to less than 35% of its maximal binding or non-competing if the signal was greater than 86%. A level of 36%–85% was considered partial competition. Thermolysin cleavage removes the epitope for most tested glycan cap antibodies that showed very low binding to Jurkat-EBOV GP_CL_. This study served as an additional control to confirm that cleavage inhibition measured as percent of RBS exposure is not due to MR78 binding completion with residually bound glycan cap antibody on Jurkat-EBOV GP_CL_.

#### Cell surface displayed GP cleavage inhibition

Jurkat-EBOV GP cells were pre-incubated with serial dilutions of mAbs in PBS for 20 min at room temperature, then incubated with thermolysin (Promega) for 20 min at 37°C. The reaction was stopped by addition of the incubation buffer containing DPBS, 2% heat inactivated FBS and 2 mM EDTA (pH 8.0). Washed cells were incubated with 5 μg/mL Alexa Fluor 657-labeled RBS-specific mAb MR78 at 4°C for 60 min. Stained cells were washed, fixed, and analyzed by flow cytometry using iQue Screener Plus flow cytometer. Cells were gated for the viable population. Background staining was determined from binding of the labeled mAb MR78 to Jurkat-EBOV GP (uncleaved) cells. Results are expressed as the percent of RBS exposure inhibition in the presence of tested mAb relative to controls for minimal binding of labeled MR78 mAb-only to intact (uncleaved) Jurkat EBOV-GP, and maximal binding of labeled MR78 mAb-only to cleaved Jurkat-EBOV GP. The GP base-directed antibody 2G4 (Qiu et al., 2011) and 2D22 served as negative controls (Fibriansah et al., 2015). BDBV-329 was excluded because it does not bind to EBOV and BDBV-43 was excluded due to poor recombinant expression of the antibody.

#### Construct design, expression, and protein purification

EBOV GP (Makona) (residues 32–644, GenBank AKG65268.1) with an N-terminal tissue plasminogen activator (*Homo sapiens*) signal sequence was codon optimized for mammalian protein expression, synthesized and subcloned into the pPPI4 expression vector (GenScript). A C-terminal enterokinase (Ek) cut site (DDDDK) was introduced after residue 628 followed by a short linker (AG) and two streptavidin tags (WSHPQFEK) separated by a GS-linker (GGGSGGGSGGGS). Residues 310–460 were removed to produce EBOV GPΔMuc. BDBV GP (residues 1–643, GenBank ALT19772.1) with the GP-associated signal peptide, an Ek cut site after residue 643 followed by an AG-linker and the double streptavidin tag as described above was codon optimized for mammalian protein expression, synthesized and subcloned into pPPI4. Residues 313–470 were removed to produce BDBV GPΔMuc. EBOV sGP (Mayinga) (residues 1–314, GenBank AAD14584.1) with the sGP-associated signal peptide an enterokinase cut site after residue 314 followed by an AG-linker and a double streptavidin tag was codon optimized for mammalian protein expression, synthesized and subcloned into pPPI4.

All GPs were expressed and purified in transiently transfected FreeStyle-293-F cells at a density of 0.8–1.5 × 10^6^ cells/mL using 750 μg of DNA and 2.25 mg of polyethylenimine “Max” (MW 25,000, Polyscience, Inc.) mixed with 50 mL of Opti-MEM (ThermoFisher Scientific). Solutions were sterile filtered using 0.22 μm Steriflip disposable filters (Millipore) and allowed to incubate at room temperature for 30 min before being added to cultures. After 5 days of expression at 37°C and 8% CO_2_, cells were harvested by centrifugation (8,000 × g for 1hr at 4°C) and filtered to remove cellular debris. BioLock biotin blocking solution (IBA Lifesciences) was added to supernatant according to the manufacturer’s protocol before being loaded onto Strep-Tactin Superflow Plus beads (QIAGEN) that had been pre-equilibrated in 1X Strep Buffer (100 mM Tris, pH 8.0, 150 mM NaCl and 1mM EDTA). Beads were washed with 10 mL of 1X Strep Buffer and GPs were eluted by addition of 2.5 mM d-desthiobiotin added to 10 mL of 1X Strep Buffer. GPs were further purified by size exclusion chromatography (SEC) using an S200 increase (S200I, GE) column equilibrated in 1X TBS (150 mM NaCl, 20 mM Tris, pH 7.4).

For EBOV-237, BDBV-329, EBOV-442, EBOV-437 and 2D22 recombinant mAb production, cDNA encoding the genes of heavy and light chains were cloned into the pTwist CMV Betaglobin WPRE Neo vector encoding IgG1 or Fab- heavy chain (McLean et al., 2000), or monocistronic expression vector pTwist-mCis_G1 (Zost et al., 2020) and transformed into *E. coli* cells. mAb proteins were produced after transient transfection of ExpiCHO cells following the manufacturer’s protocol and were purified from filtered culture supernatants by fast protein liquid chromatography (FPLC) on an AKTA instrument using HiTrap MabSelect Sure column for IgG (GE Healthcare Life Sciences) or CaptureSelect IgG-CH1 column for Fab (ThermoFisher Scientific). Purified mAbs were buffer exchanged into PBS, filtered using sterile 0.45 μm pore size filter devices (Millipore), concentrated, and stored in aliquots at −80°C until use.

For BDBV-289, BDBV-43, EBOV-293 and EBOV-296 antibody expression, sequences were optimized for mammalian expression, synthesized and subcloned into the expression vector AbVec containing the human IgG HC constant region or the human lambda or kappa LC constant region (GenScript). Fab was produced by the introduction of a stop codon after residue 226 in the HC hinge-region. ADI-15878 Fab and ADI-16061 Fab were used as a fiducials in this study and were produced as previously described (Murin et al., 2018). IgGs and Fab were transiently transfected as described above for GPs, except that 500 μg of HC DNA and 250 μg of LC DNA was mixed to encourage HC/LC pairing and the avoidance of LC dimers. For BDBV-289 and BDBV-43 Fab, cell supernatants were loaded onto 5 mL Lambda (BDBV-289) or Kappa (BDBV-43) Select columns (GE) that had been equilibrated in 1X phosphate buffered saline (PBS, QualityBiological) followed by elution with 0.1 M glycine, pH 3.0. Fab were subsequently buffer exchanged into 20 mM sodium acetate (NaOAc), pH 5.6 by dialysis and loaded onto a MonoS column (GE). Fab were then eluted with a gradient of 1M KCl. For EBOV-437, EBOV-442, EBOV-293 and EBOV-296 Fab, cell supernatants were loaded onto a 1 mL or 5 mL Capture Select column (ThermoFisher Scientific) and eluted with 0.1 M glycine, pH 3.0. Appropriate fractions were pooled and further purified by SEC using an S200I column equilibrated in 1X TBS buffer. For IgG, supernatants were loaded onto a HiTrap 5 mL mAb Select column (GE) that had been pre-equilibrated in 1X PBS followed by elution with 0.1 M glycine, pH 3.0 and neutralization with 1M Tris, pH 8.5. Appropriate fractions were pooled and further purified by SEC using an S200I column that had been equilibrated with 1X TBS.

#### Crystallization and Structure Determination of BDBV-289 Fab

Fabs produced for crystallographic studies were made in Expi-CHO cells per the manufacturer’s “max titer” protocol (GIBCO/ThermoFisher Scientific) and purified as described above. BDBV-289 Fab was screened for crystallization using the Joint Center for Structural Genomics (JCSG) Rigaku CrystalMation at The Scripps Research Institute against the JCSG Core Suites I-IV. Protein at 7.4 mg/mL was mixed 1:1 with precipitants and crystallized using the vapor diffusion method at both room temperature and 4°C. Crystals grew in 0.1M HEPES pH 6.5 and 20% (w/v) polyethylene glycol 6000 at 4°C. Crystals were cryoprotected with well solution augmented with 30% ethylene glycol. Data were collected at Stanford Synchrotron Radiation Light Source beamline 12–2. Data were indexed, integrated and scaled using HKL-2000 (Otwinowski and Minor, 1997) to 3.0 Å (Table S2). Crystals belonged to the space group P6_1_ with a single Fab in the asymmetric unit.

Data were phased using Phaser (McCoy et al., 2007) with molecular replacement by a homology model generated using Swiss Modeler (Biasini et al., 2014). A single Fab was placed in the asymmetric unit. This initial solution was rebuilt manually in Coot (Emsley et al., 2010), followed by multiple rounds of refinement in Phenex.refine (Adams et al., 2010) and model building with Coot. Translation/Libration/Screw (TLS) groups were introduced toward the end of refinement. Four TLS groups were set manually with one for each immunoglobulin domain. A large positive density seen in the difference map was modeled as PEG after evaluating fits for all components of the buffer.

#### Cryo-EM sample preparation

EBOV/Mak GPΔMuc was incubated overnight with a 5-fold molar excess of each Fab at 4°C. The complexes were then purified by SEC using an S200I column equilibrated in 1X TBS and concentrated using a 100-kDa concentrator (Amicon Ultra, Millipore) and mixed with detergent immediately prior to freezing (Table S3). Vitrification was performed with a Vitrobot (ThermoFisher Scientific) equilibrated to 4°C and 100% humidity. Cryo-EM grids were plasma cleaned for 5 s using a mixture of Ar/O_2_ (Gatan Solarus 950 Plasma system) followed by blotting on both sides of the grid with filter paper (Whatman No. 1). See Table S3 for additional details for individual complexes. Note that ADI-15878 Fab was added to the BDBV-43 complex and ADI-16061 Fab and EBOV-515 Fab was added to the EBOV-437, EBOV-442, EBOV-293, EBOV-296, EBOV-237 and BDBV-329 complexes to assist in angular sampling and orientations in ice.

#### Cryo-EM data collection and processing

Cryo-EM data were collected according to Table S1. Micrographs were aligned and dose-weighted using MotionCor2 (Zheng et al., 2017). CTF estimation was completed using GCTF (Zhang, 2016). Particle picking and initial 2D classification were initially performed using CryoSPARC 2.0 (Punjani et al., 2017) to clean up particle stacks and exclude any complexes that were degrading. For those reconstructions that required more extensive 3D classification, particle picks were then imported into Relion 3.1b1 (Zivanov et al., 2018) for 3D classification and then refinement using appropriate symmetry where necessary and a tight mask around the GP/Fab complex of interest. CTF refinement was then performed by either Relion or CryoSPARC to increase map quality and resolution. There was no density for ADI-16061 in any of the maps and we did not build density for ADI-15878 in the BDBV-43 map (this was previously deposited under PDB 6DZM). We chose not to build a model into EBOV-515 density but included this density in our reconstructions to assist with particle alignment.

#### Cryo-EM model building and refinement

Homology models of Fab were first generated using SWISS-MODEL (Biasini et al., 2014). Models of BDBV GP (PDB: 6DZM) and EBOV GP (PDB: 5JQ3) were then added to generate starting models used for refinement. Models were fit into maps using UCSF Chimera (Pettersen et al., 2004) and refined initially using Phenix real-space refinement using NCS constraints (Liebschner et al., 2019). The refined model was then used as a template for relaxed refinement in Rosetta (DiMaio et al., 2015). The top five models were then evaluated for fit in EM density and adjusted manually using Coot (Emsley et al., 2010) to maximize fit. Finally, Man9 glycans were fit into glycan densities, trimmed and then a final refinement was performed in Rosetta. The final structures were evaluated using EMRinger (Barad et al., 2015) and Molprobity from Phenix. All figures were generated in UCSF Chimera (Pettersen et al., 2004). Antibody contacts were analyzed using LigPlot (Laskowski and Swindells, 2011), Arpeggio (Jubb et al., 2017) and UCSF Chimera (Pettersen et al., 2004).

#### Inferred germline antibody analysis

Inferred germline sequences for BDBV289 and BDBV43 F_V_ domains were determined using IMGT/V-QUEST (Brochet et al., 2008; Giudicelli et al., 2011). Nucleotide sequences of B cells originally isolated from donors were kindly provided by James Crowe and used to derive a list of likely germline VDJ genes. Those with the highest confidence were then used to reconstruct an inferred germline sequence. The mature CDRH3 sequence was included in the reconstructed germline sequences due to low confidence in predicting germline CDRH3 sequences, although some residues were predicted to be different from the germline CDRH3. For BDBV-289 and BDBV-43, inferred germline sequences were then codon optimized for mammalian protein expression and sub-cloned into the appropriate AbVec expression vector. Stop codons were introduced as described above to produce Fab.

### QUANTIFICATION AND STATISTICAL ANALYSIS

The descriptive statistics mean ± SD were determined for continuous variables as noted. EC_50_ values for mAb binding were determined after log transformation of antibody concentration using sigmoidal dose-response nonlinear regression analysis. Correlation between antibody synergy and percent monomer in GP trimer fraction was estimated using linear regression analysis. In neutralization assays, IC_50_ values were calculated after log transformation of antibody concentrations using a 4-parameter nonlinear fit analysis. Technical and biological replicates are indicated in the figure legends. Statistical analyses were performed using Prism v8 (GraphPad).

## Supplementary Material

1

2

## Figures and Tables

**KEY RESOURCES TABLE:** 

REAGENT or RESOURCE	SOURCE	IDENTIFIER
Antibodies		

BDBV-289 (recombinant IgG1)	Flyak et al., 2016; J.E. Crowe	N/A
BDBV-43 (recombinant IgG1)	Flyak et al., 2016; J.E. Crowe	N/A
EBOV-293 (recombinant IgG1)	This study	N/A
EBOV-296 (recombinant IgG1)	This study	N/A
EBOV-437 (recombinant IgG1)	Gilchuk et al., 2018; J.E. Crowe	N/A
EBOV-442 (recombinant IgG1)	Gilchuk et al., 2018; J.E. Crowe	N/A
BDBV-329 (recombinant IgG1)	Flyak et al., 2016; J.E. Crowe	N/A
EBOV-237 (recombinant IgG1)	Williamson et al., 2019. J.E. Crowe.	N/A
EBOV-515 (recombinant IgG1)	Gilchuk et al., 2018; J.E. Crowe	N/A
EBOV-520 (recombinant IgG1)	Gilchuk et al., 2018; Gilchuk et al., 2020; J.E. Crowe	N/A
ADI-15878 (recombinant IgG1)	Bornholdt et al., 2016b; Mappbio	N/A
ADI-16061 (recombinant IgG1)	Bornholdt et al., 2016b; Mappbio	N/A
2D22 (recombinant IgG1)	Fibriansah et al., 2015	N/A
2G4 (recombinant IgG1)	Qiu et al., 2011	N/A
BDBV223 (hybridoma IgG3)	Flyak et al., 2018	N/A
Goat anti-human IgG-HRP	Southern Biotech	RRID: AB_2795644
Goat anti-human IgG Fc-PE, multi-species adsorbed	Southern Biotech	RRID: AB_2795582
Goat anti-human IgG-Alexa Fluor 488	Jackson ImmunoResearch Laboratories	RRID: AB_2337831
MR78-Alexa Fluor 647	Flyak et al., 2015; Gilchuk et al., 2018	N/A

Biological samples		

Human PBMCs	Nashville Red Cross	N/A

Bacterial and viral strains		

EBOV-MA	Bray et al., 1998	N/A

Chemicals, peptides, and recombinant proteins		

FreeStyle 293 expression medium	Thermo Fisher	Cat no. 12338018
ExpiCHO expression medium	Thermo Fisher	Cat no. A2910001
ExpiCHO feed	Thermo Fisher	Cat no. A2910002
Opti-MEM	Thermo Fisher	Cat no. 31985070
Polyethylenimine “Max”	Polyscience, Inc.	Cat no. 24765-1
BioLock	IBA Lifesciences	Cat no. 2-0205-250
d-desthiobiotin	Sigma	D1411
EBOV GPΔTM-Makona variant	This study	N/A
EBOV GPΔMuc-Makona variant	This study	N/A
EBOV GPΔMuc-Mayinga variant	This study	N/A
EBOV sGP	This study	N/A
BDBV GPΔTM	This study	N/A
BDBV GPΔMuc	This study	N/A
SUDV GPΔTM	This study	N/A
n-Dodecyl-beta-Maltoside	Anatrace	Part no. D310S
A8–35 amphipole	Anatrace	Part no. A835
Fluorinated octyl maltoside	Anatrace	Part no. O310F

Deposited data		

BDBV-289 Fab complex with EBOV GPΔMuc-Makona strain model	This paper	PDB: 7KEL
BDBV-43 Fab and ADI-15878 Fab complex with BDBV GPΔMuc cryo-EM map	This paper	EMDB: EMD-22841
BDBV-43 Fab complex with BDBV GPΔMuc model	This paper	PDB: 7KEW
EBOV-293 Fab and EBOV-515 Fab complex with EBOV GPΔMuc-Makona strain cryo-EM map	This paper	EMDB: EMD-22842
EBOV-293 Fab complex with EBOV GPΔMuc-Makona strain model	This paper	PDB: 7KEX
EBOV-296 Fab and EBOV-515 Fab complex with EBOV GPΔMuc-Makona strain cryo-EM map	This paper	EMDB: EMD-22847
EBOV-296 Fab complex with EBOV GPΔMuc-Makona strain model	This paper	PDB: 7KF9
EBOV-437 Fab and EBOV-515 Fab complex with EBOV GPΔMuc-Makona strain cryo-EM map	This paper	EMDB: EMD-22853
EBOV-437 Fab complex with EBOV GPΔMuc-Makona strain model	This paper	PDB: 7KFH
EBOV-442 Fab and EBOV-515 Fab complex with EBOV GPΔMuc-Makona strain cryo-EM map	This paper	EMDB: EMD-22848
EBOV-442 Fab complex with EBOV GPΔMuc-Makona strain model	This paper	PDB: 7KFB
BDBV-329 Fab and EBOV-515 Fab complex with BDBV GPΔMuc cryo-EM map	This paper	EMDB: EMD-22851
BDBV-329 Fab complex with BDBV GPΔMuc model	This paper	PDB: 7KFE
EBOV-237 Fab and EBOV-515 Fab complex with EBOV GPΔMuc-Makona strain cryo-EM map	This paper	EMDB: EMD-22852
BDBV-289 Fab crystal structure	This paper	PDB: 7KFG

Experimental models: Cell lines		

Human: FreeStyle 293-F	Thermo Fisher	R79007
Human: HEK293T	ATCC	CRL-3216
HeLa S3	ATCC	CCL-2.2
Human: ExpiCHO	Thermo Fisher	A29127
Human: Jurkat E6–1	ATCC	TIB-152
Human: Jurkat-EBOV GP	Davis et al., 2019; Gilchuk et al., 2018	N/A
Human: HMMA2.5 myeloma	Yu et al., 2008	N/A

Experimental models: Organisms/strains		

Mouse: Female BALB/c	Jackson Laboratory	Stock no. 00651

Recombinant DNA		

pPPI4-EBOV GPΔMuc Makona-Ek-ddStrep	This study	N/A
pPPI4-BDBV GPΔMuc-Ek-ddStrep	This study	N/A
AbVec-BDBV-289 heavy chain IgG	This study	N/A
AbVec-BDBV-289 heavy chain Fab	This study	N/A
AbVec-BDBV-289 light chain lambda	This study	N/A
AbVec-BDBV-43 heavy chain IgG	This study	N/A
AbVec-BDBV-43 heavy chain Fab	This study	N/A
AbVec-BDBV-43 light chain kappa	This study	N/A
AbVec-BDBV-293 heavy chain IgG	This study	N/A
AbVec-BDBV-293 heavy chain Fab	This study	N/A
AbVec-BDBV-293 light chain lambda	This study	N/A
AbVec-BDBV-296 heavy chain IgG	This study	N/A
AbVec-BDBV-296 heavy chain Fab	This study	N/A
AbVec-BDBV-296 light chain lambda	This study	N/A
AbVec-ADI-15878 heavy chain Fab	Murin et al., 2018	N/A
AbVec-ADI-15878 light chain kappa	Murin et al., 2018	N/A
AbVec-ADI-16061 heavy chain Fab	Murin et al., 2018	N/A
AbVec-ADI-16061 light chain kappa	Murin et al., 2018	N/A
pTwist-mCis_G1-EBOV-437 IgG	This study	N/A
pTwist-mCis_G1-EBOV-437 Fab	This study	N/A
pTwist-mCis_G1-EBOV-442 IgG	This study	N/A
pTwist-mCis_G1-EBOV-442 Fab	This study	N/A
pTwist CMV Betaglobin WPRE Neo-BDBV-329 heavy chain IgG	This study	N/A
pTwist CMV Betaglobin WPRE Neo-BDBV-329 heavy chain Fab	This study	N/A
pTwist CMV Betaglobin WPRE Neo-BDBV-329 light chain	This study	N/A
pTwist CMV Betaglobin WPRE Neo-EBOV-237 heavy chain IgG	This study	N/A
pTwist CMV Betaglobin WPRE Neo-EBOV-237 heavy chain Fab	This study	N/A
pTwist CMV Betaglobin WPRE Neo-EBOV-237 light chain	This study	N/A
pTwist-mCis_G1-MR78 IgG	This study	N/A
pTwist CMV Betaglobin WPRE Neo-DENV-2D22 heavy chain IgG	This study	N/A
pTwist CMV Betaglobin WPRE Neo-DENV-2D22 light chain	This study	N/A
pTwist CMV Betaglobin WPRE Neo-2G4 heavy chain IgG	This study	N/A
pTwist CMV Betaglobin WPRE Neo-2G4 light chain	This study	N/A

Software and algorithms		

COOT	Emsley et al., 2010	https://www2.mrc-lmb.cam.ac.uk/personal/pemsley/coot
Phenix	Adams et al., 2010	https://www.phenix-online.org
HKL2000	Otwinowski and Minor, 1997	https://www.hkl-xray.com/
MotionCor2	Zheng et al., 2017	https://msg.ucsf.edu/em/software/motioncor2.html
GCTF	Zhang, 2016	N/A
DoG Picker	Voss et al., 2009	https://emg.nysbc.org/redmine/projects/software/wiki/DoGpicker
Relion 3.1b1	Zivanov et al., 2018	https://www2.mrc-lmb.cam.ac.uk/relion/index.php/Main_Page
CryoSPARC 2.0	Punjani et al., 2017	https://cryosparc.com/
UCSF Chimera	Pettersen et al., 2004	https://www.cgl.ucsf.edu/chimera/
Rosetta	DiMaio et al., 2015	https://www.rosettacommons.org/
Swiss Modeler	Biasini et al., 2014	https://swissmodel.expasy.org/
Phaser	McCoy et al., 2007	https://www.ccp4.ac.uk/html/phaser.html
EMRinger	Barad et al., 2015	https://fraserlab.com/2015/02/18/EMringer/
Molprobity	Chen et al., 2010	http://molprobity.biochem.duke.edu
LigPlot	https://www.ebi.ac.uk/thorntonsrv/software/LIGPLOT/
Arpeggio	Jubb et al., 2017	http://biosig.unimelb.edu.au/arpeggioweb/
ForeCyt Standard 6.2 (R1)	Intellicyt	https://intellicyt.com/products/software/
GraphPad Prism 8.4.3		https://www.graphpad.com/

Other		

ABI3700 automated DNA sequencer	Applied Biosystems	https://www.thermofisher.com/us/en/home.html
iQue Screener Plus flow cytometer	Intellicyt	https://intellicyt.com/
Spectrophotometer	Biotek	https://www.biotek.com/
Titan Krios 300kV electron microscope	Thermo Fisher	https://www.fei.com/
Talos Arctica 200kV electron microscope	Thermo Fisher	https://www.fei.com/
K2 Summit camera	Gatan	https://www.gatan.com
Vitrobot	Thermo Fisher	https://www.fei.com/
Gatan Solarus 950 Plasma system	Gatan	https://www.gatan.com
Quantifoil R 1.2/1.3 holey carbon EM grids	Electron Microscopy Services	https://www.emsdiasum.com/microscopy/products/grids/quantifoil.aspx

## Highlights

- Glycan cap antibody-mediated GP destabilization correlates with synergy
- Cryo-EM structures reveal antibodies target a highly conserved epitope
- Antibodies use long CDRH3 loops to displace and mimic portions of the glycan cap
- Glycan cap antibodies block cleavage events required for viral entry
