# Supplementary material for: Convergence of a common solution for broad ebolavirus neutralization by glycan cap-directed human antibodies
Source: Cell Rep. Author manuscript; Available in PMC 2021 May 19. (PMC8133395; doi:10.1016/j.celrep.2021.108984)
Supplement: 1 [file NIHMS1693704-supplement-1.pdf]

Cell Reports, Volume *35*

Supplemental information

Convergence of a common solution for broad ebolavirus neutralization by glycan cap-directed human antibodies

Charles D. Murin, Pavlo Gilchuk, Philipp A. Ilinykh, Kai Huang, Natalia Kuzmina, Xiaoli Shen, Jessica F. Bruhn, Aubrey L. Bryan, Edgar Davidson, Benjamin J. Doranz, Lauren E. Williamson, Jeffrey Copps, Tanwee Alkutkar, Andrew I. Flyak, Alexander Bukreyev, James E. Crowe Jr., and Andrew B. Ward

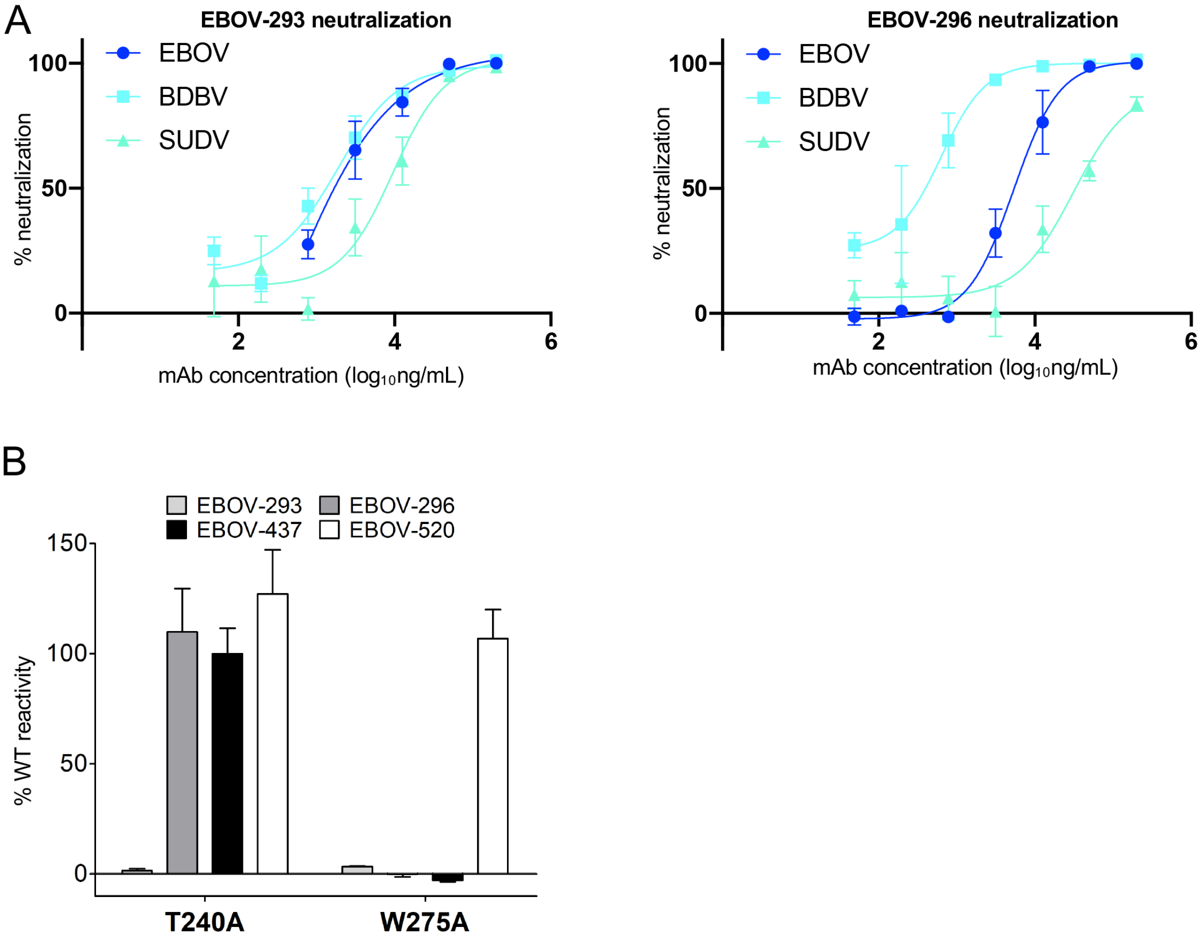

Figure S1. Characterization of EBOV-293 and EBOV-296 antibodies from a human individual, related to Figure 1 (see also Table S1). (**A**) Neutralizing data for EBOV-293 and EBOV-293 (hybridoma-derived antibodies) for the three major ebolaviruses. IC_{50} values are listed in Table 1. (**B**) Site-directed mutagenesis of residues determined by alanine scanning to be important for the binding of EBOV-293 and EBOV-296. EBOV-437 and EBOV-520 are included for reference and as controls.

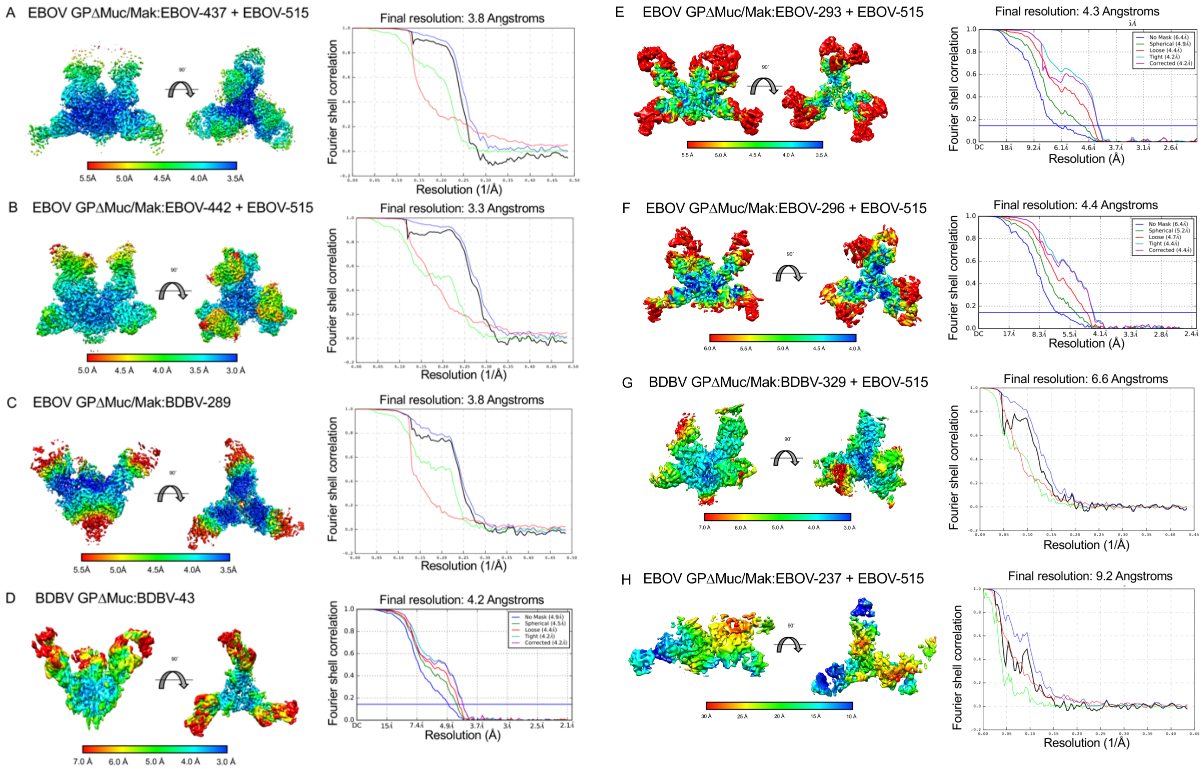

Figure S2. Cryo-EM local resolution estimation and FSC curves, related to Figure 2. Local resolution is plotted onto the cryo-EM maps of structures solved in this study according to the scale for each figure. Side views (left) and top views (right) are shown, with respect to the viral membrane. Fourier shell correlation curves for each reconstruction are shown with the estimated resolution indicated. Relion 3.1 was used in panels A to C and G to H. Cryosparc 2.0 was used for reconstructions in panels D to F.

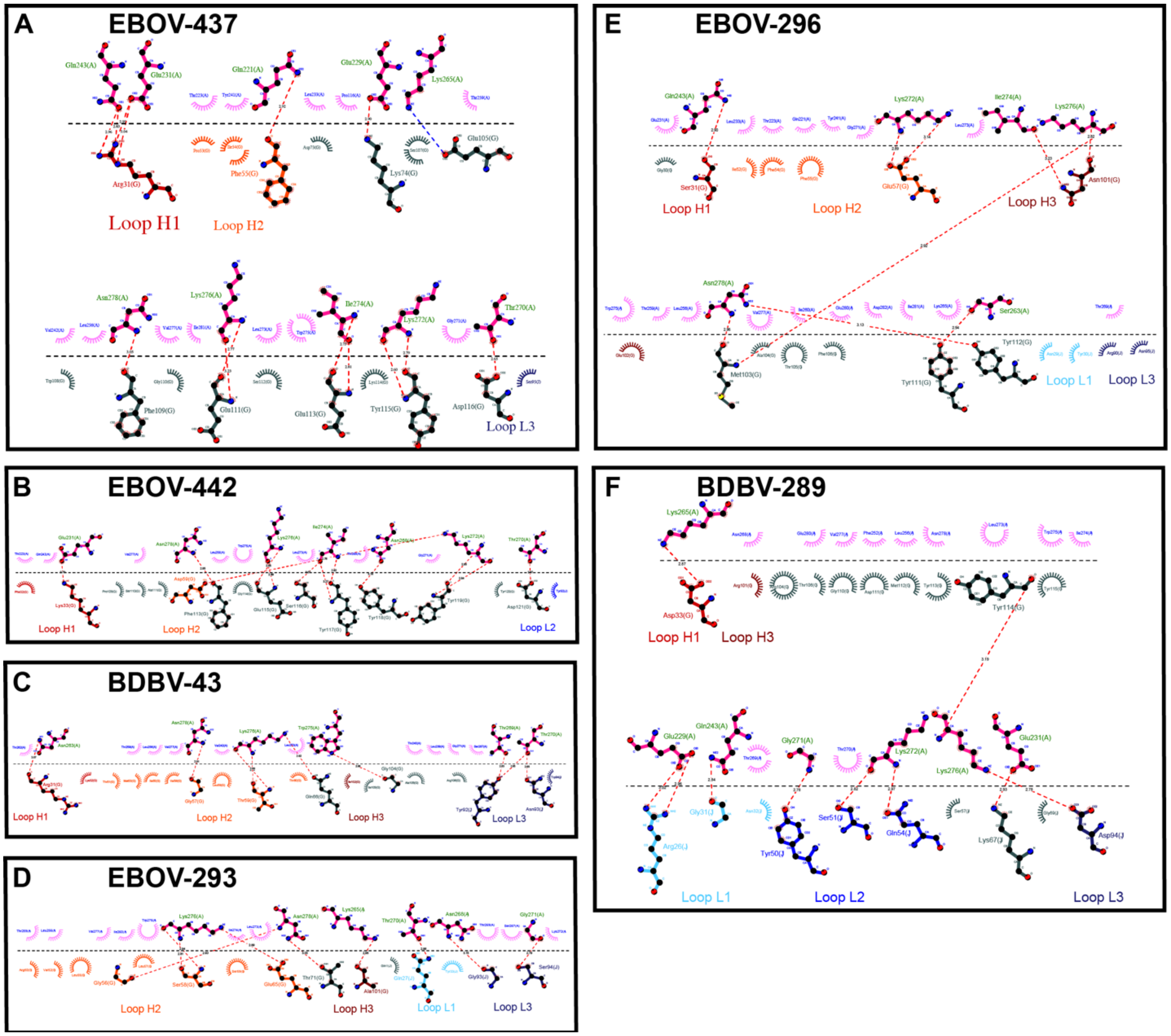

Figure S3. Glycan cap antibody epitope and paratope interactions, related to Figure 3. LigPlot schematics (**A to F**) for each of the glycan cap antibody structures solved in this study are shown. Residues are labeled with their corresponding chain number in parentheses. Glycoprotein residues involved in contacts are shown above the dashed black line and those from antibodies are shown below the dashed line. Liganded residues involved in hydrogen bonds are rendered as ball-and-stick (with individual atoms labeled), and hydrogen bonds are indicated by red dashed lines with the bond length shown in Angstroms. Symbols of arcs with rays indicate residues involved in hydrophobic interactions and point in the direction the interaction takes place. Atoms with hydrophobic interactions are also indicated with rays.

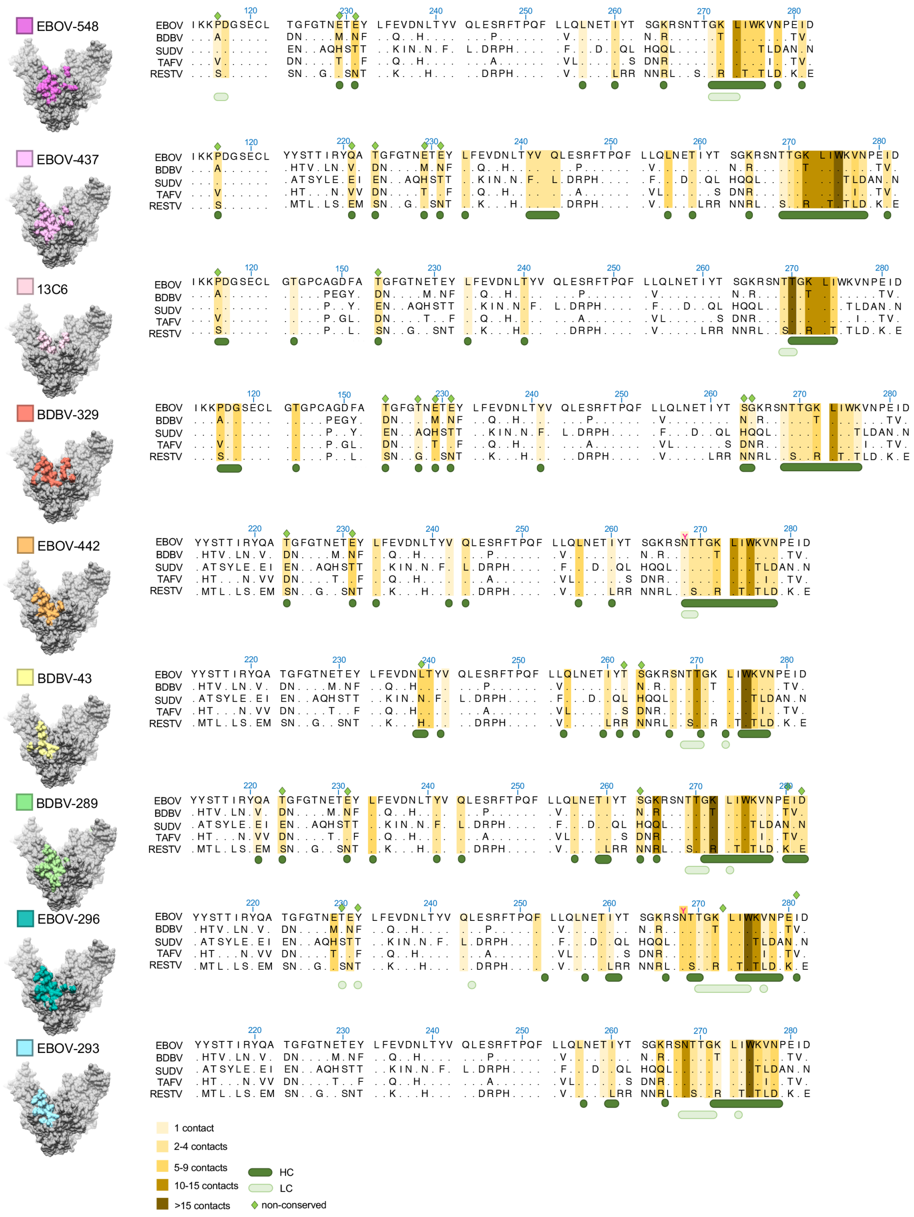

Figure S4. Epitope conservation of glycan cap antibodies, related to Figures 2 and 3. Apo-GP is rendered in grey on the left with the corresponding glycan cap antibody footprint highlighted. On the right are aligned sequences of the interacting regions on GP. Total contacts for each residue at 4 Å distance or less were determined (see Table S4) and residues are highlighted in gold according to the number of contacts, with darker brown indicating more contacts and thus more likely to be an important residue for binding. Residues that are not well-conserved are marked with a green diamond. HC contacts are indicated below in dark green and LC contacts in light green.

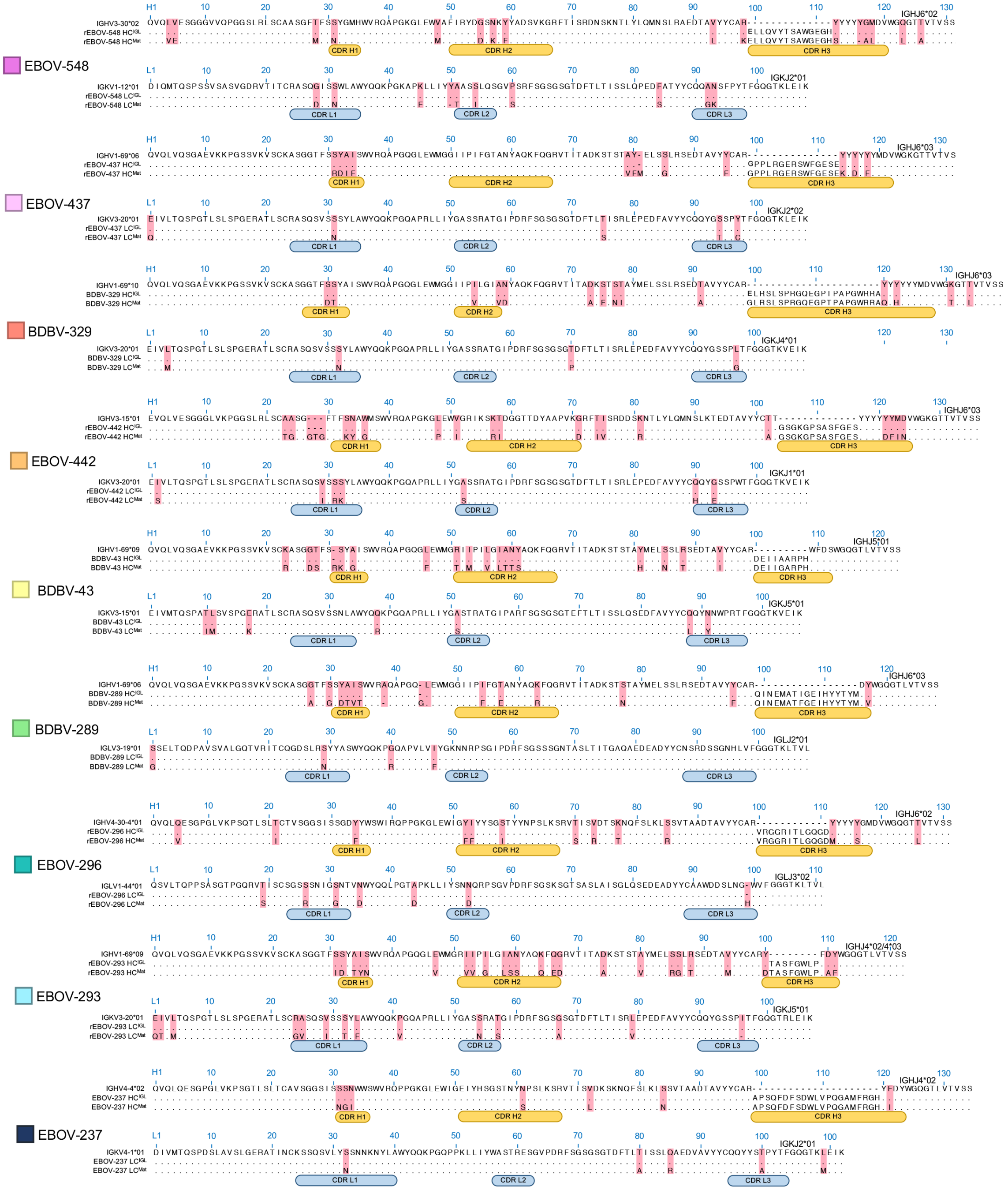

Figure S5. Inferred germline sequences of glycan cap antibodies, related to figures 4 and 5. The germline genes for each glycan cap antibody were determined (top line) from nucleotide sequences (see methods) and inferred germline (IGL) sequences for the HC and LC were assembled (middle line) and aligned with the mature (Mat) sequence (bottom line). HC CDR loops are indicated below in yellow and LC CDR loops are indicated in blue. Residues that changed from germline are highlighted in pink.

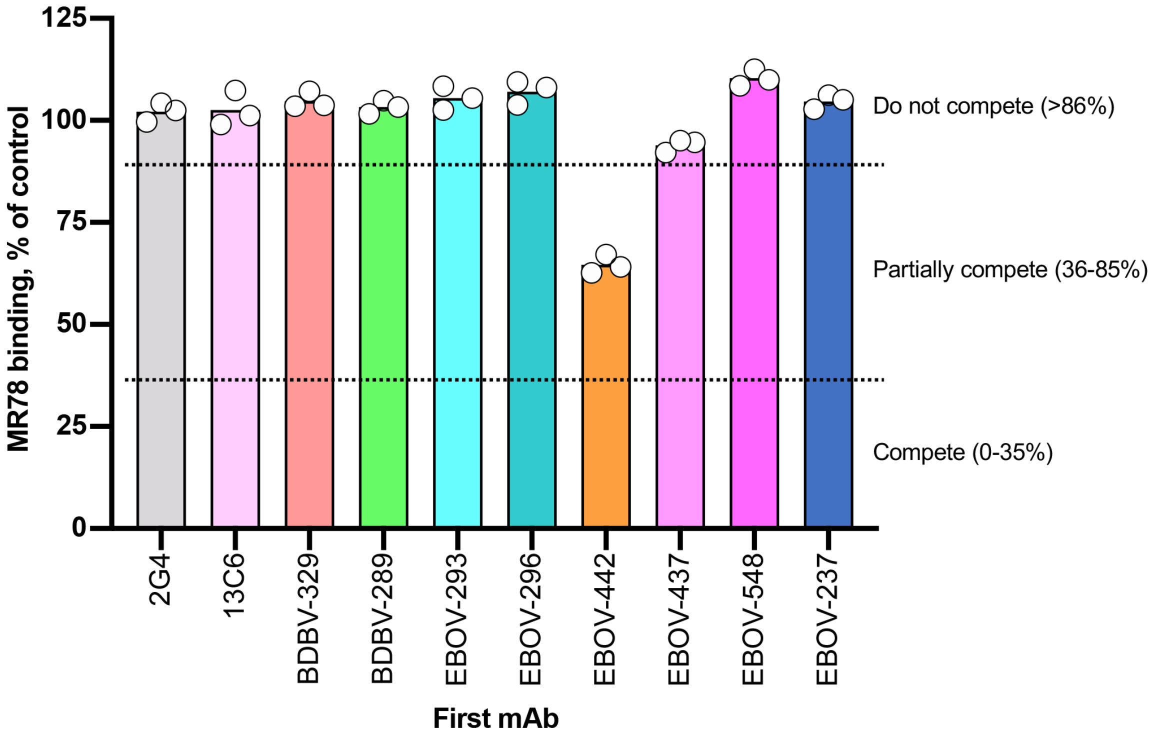

Figure S6. Control for specificity of RBS exposure measurement on Jurkat-EBOV GP_{CL} in a presence of glycan cap antibody, related to Figure 7. Jurkat-EBOV GP_{CL} cells that obtained after thermolysin treatment of Jurkat-EBOV cells were pre-incubated with 20 µg/mL of respective glycan cap antibody followed by addition of fluorescently labeled antibody MR78. Binding was analyzed by flow cytometry, and the results are expressed as the percent of binding in the presence of glycan cap mAb relative to MR78 alone (maximal binding) minus background. The antibodies were considered competing if the presence of first antibody reduced the signal of the second antibody to less than 35% of its maximal binding or non-competing if the signal was greater than 86%. A level of 36–85% was considered partial competition. It should be noted that thermolysin cleavage removes the epitope for most tested glycan cap antibodies that showed very low specific binding to Jurkat-EBOV GP_{CL}.

Antibody	GP binding by ELISA, EC_{50} (ng/mL)				Virus neutralization, IC_{50} (ng/mL)			Site on GP	GP mutations	IgG therapy in mice (EBOV), % survival	Antibody source
	**EBOV GP**	**BDBV GP**	**SUDV GP**	**EBOV sGP***	**EBOV**	**BDBV**	**SUDV**				
EBOV-293	49	198	91	3	1,672	1,892	10,456	GC	T240A, W275A	100*	This study
EBOV-296	39	78	514	4	5,758	534	39,217	GC	W275A	20*	This study
BDBV-329	>	135	>	21 (BDBV sGP)	>	770	>	GC	ND	ND	Flyak *et al.* 2016
BDBV-289	29	20	103	1	602	32	>	GC	W275A, Y241A	80	Flyak *et al.* 2016
BDBV-43	22	29	21	18 (BDBV sGP)	7,248	139	318	GC	L273P	20	Flyak *et al.* 2016
EBOV-237	25	3,367	>	162	780	ND	ND	GC	I260R, N278A, P279A, S322G	80-90**	Williamson *et al.* 2019
EBOV-437	3	11	4	1	8,660***	>	>	GC	W275A	ND	Gilchuk *et al.* 2018
EBOV-442	1	3	6	1	467	1,489	<75%***	GC	W275A, L273P	80	Gilchuk *et al.* 2018
EBOV-548	6	7	132	1	1,601	2,262	<12%***	GC	T240A, R266A, T269A, T270A, I274A, W275	40	Gilchuk *et al.* 2020
13C6	20	>	>	2	>	>	>	GC	T270A, K272A, T240N	100	Wilson *et al.* 2000
2G4	203	>	>	>	139	>	>	Base	C511A, G553A, N550A, D552A, C556A, Q508R	60	Qiu *et al.* 2012

Table S1. **Summary of binding and functional activities of characterized antibodies, related to Figure 1.** Characteristics for previously described antibodies were included for comparative purposes.*Determined in this study; ** prophylaxis efficacy; ***incomplete neutralization at highest tested Ab concentration (0.2 mg/mL); ">" indicates activity was not detected at highest tested Ab concentration; ND – not determined.

Antibody	Subject ID	VL	JL	VH	D	JH	CDRH3 length (amino acids)	Heavy chain VH mutations (%)*	Light chain VL mutations (%)**
EBOV-293	859	*IGKV3-20*01*	*IGKJ5*01*	*IGHV1-69*09*	*IGHD3-9*01*	*IGHJ4*02/4*03*	21	24/122 (19.6)	14/107 (13)
EBOV-296	859	*IGLV1-44*01*	*IGLJ3*02*	*IGHV4-30-4*01*	*IGHD3-10*01*	*IGHJ6*02*	21	13/131 (9.9)	7/111 (6.3)
BDBV-329	3	*IGKV3-20*01*	*IGKJ4*01*	*IGHV1-69*10*	*IGHD1-26*	*IGHJ6*03*	33	14/139 (10)	4/108 (3.7)
BDBV-289	7	*IGLV3-19*01*	*IGLJ2*01*	*IGHV1-69*06*	*IGHD5-24*	*IGHJ6*03*	21	15/129 (11.6)	4/108 (3.7)
BDBV-43	7	*IGKV3-15*01*	*IGKJ5*01*	*IGHV1-69*09*	*IGHD6-6*	*IGHJ5*01*	15	18/123 (14.6)	7/106 (6.6)
EBOV-237	EVD5	*IGKV4-1*01*	*IGKJ2*01*	*IGHV4-4*02*	*IGHD3-3*01*	*IGHJ4*02*	25	7/134 (5.2)	5/102 (4.9)
EBOV-437	963	*IGKV3-20*01*	*IGKJ2*02*	*IGHV1-69*06*	*IGHD3-10*01*	*IGHJ6*03*	24	12/132 (9)	5/108 (4)
EBOV-442	963	*IGKV3-20*01*	*IGKJ1*01*	*IGHV3-15*01*	*IGHD3-16*01*	*IGHJ6*03*	22	21/136 (15)	7/108 (6.4)
EBOV-548	963	*IGKV1-12*01*	*IGKJ2*01*	*IGHV3-30*02*	*IGHD6-19*01*	*IGHJ6*02*	22	16/131 (12)	10/108 (9.2)

Table S2. Summary of antibody germline characteristics, related to Figure 1. *Heavy chain number of mutations in VH-encoded region from germline per total amino acids (%); **Light chain number of mutations in VL from germline per total amino acids (%)

Map	EBOV GPdMuc:BDBV289	BDBV GPdMuc:BDBV43 + ADI-15878	EBOV GPdMuc:EBOV-293 + EBOV-515	EBOV GPdMuc:EBOV-296 + EBOV-515	EBOV GPdMuc:EBOV-437 + EBOV-515	EBOV GPdMuc:EBOV-442 + EBOV-515	BDBV GPdMuc:BDBV329 + EBOV-515	EBOV GPdMuc:EBOV-237 + EBOV-515
**Data collection**	18jan23c	17sep13b	19aug29a	19aug28b	19aug24a	19aug23b	19nov11g	19nov12i
Microscope	Titan Krios	Titan Krios	Talos Arctica	Titon Krios	Titan Krios	Titan Krios	Talos Arctica	Talos Arctica
Voltage (kV)	300	300	200	300	300	300	200	200
Detector	K2 Summit	K2 Summit	K2 Summit	K2 Summit	K2 Summit	K2 Summit	K2 Summit	K2 Summit
Recording mode	Counting	Counting	Counting	Counting	Counting	Counting	Counting	Counting
Magnification	48,543	48,543	43,478	48,543	48,543	48,543	43,478	43,478
Movie micrograph pixel size (Å)	1.03	1.03	1.15	1.03	1.03	1.03	1.15	1.15
Dose rate (e-/[(camera pixel)*s])	7.5	7.5	6.25	6.43	6.43	6.43	6.25	6.25
Number of frames per movie	32	32	50	42	42	42	42	42
Frame exposure time (ms)	250	250	250	250	250	250	250	250
Movie micrograph exposure time (s)	8	7.5	10.5	10.5	10.5	10.5	10.5	10.5
Total dose (e-/Å^2)	60	60	50	50	50	50	50	50
Defocus range (µm)	-1 to -3.5	-1 to -3.5	1 to -3.5	-1 to -3.5	-1 to -3.5	-1 to -3.5	-1 to -3.5	-1 to -3.5
**EM data processing**	-	-	-	-	-	-	-	-
Software used	Relion 3.1b	CryoSPARC 2.0	CryoSPARC 2.0	CryoSPARC 2.0	Relion 3.1b	Relion 3.1b	Relion 3.1b	Relion 3.1b
Number of movie micrographs	1,061	2,753	796	785	1,521	1,020	1,090	844
Number of particles in map	32,108	383,783	79,722	69,564	42,770	21,285	45,640	17,244
Symmetry	C3	C3	C3	C3	C3	C3	C1	C1
Map resolution (FSC 0.143;Å)	3.27	4.1	4.5	4.3	3.4	3.2	4.7	9.2
Map sharpening B-factor (Å^2)	-137	-186	-216	-126	-142	-92	-151	-369
**Structure building and validation**	-	-	-	-	-	-	-	-
Number of atoms in deposited model	13,790	13,173	18,309	18,570	18,981	18,783		N/A
GP1	5,682	5,628	5,352	5,298	5,589	5,541		N/A
GP2	2,682	2,529	2,343	2,388	2.454	2,373		N/A
BDBV289	5,426	-	-	-	-	-	-	-
BDBV43	-	5,361	-	-	-	-	-	-
EBOV-293	-	-	5,211	-	-	-		-
EBOV-296	-	-	-	5,481	-	-		-
EBOV-437	-	-	-	-	5,535	-	-	-
EBOV-442	-	-		-	-	5,571	-	-
BDBV-329	-	-	-		-	-	-	-
EBOV-515	-	-	5,403	5,403	5,403	5,298	-	-
glycans	309	309	351	309	393	534		N/A
MolProbity score	0.62	0.92	1.07	0.96	0.76	1.02		N/A
Clashscore	0.33	0.54	0.97	0.71	0.35	1.84		N/A
EMRinger score	4.46	1.04	1.06	0.93	3.44	3.62		N/A
RMSD from ideal	-	-	-	-	-	-		-
Bond length (Å)	0.02	0.021	0.021	0.021	0.021	0.021		N/A
Bond angle (degrees)	1.65	1.81	1.82	1.81	1.71	1.8		N/A
Ramachandran plot	-	-	-	-	-	-		-
Favored (%)	99.65	96.47	95.91	96.45	97.32	97.65		N/A
Allowed (%)	0.35	3.16	3.56	3.42	2.17	1.69		N/A
Outliers (%)	0	0.37	0.53	0.13	0.51	0.65		N/A
Average B-factor	61.07	160.49	191.2	201.24	144.66	127.53		N/A

Table S3. Cryo-EM data collection and validation statistics, related to Figure 2 and Figure 3.

Residue number	Total Contacts	Residue	Number of amino acid contacts with GP								
			EBOV-548	EBOV-437	13C6	BDBV-329	EBOV-442	BDBV-43	BDBV289	EBOV-296	EBOV-293
116	13	P	1	3	4	5					
117	5	D	3		1	1					
118	5	G				5					
144	7	T			1	6					
221	4	Q		3					1		
222	0	A									
223	15	T		4	3	2	3		3		
227	1	T				1					
229	18	E	2	2		7				7	
231	26	E	7	4		2	8		3	2	
233	13	L		2	1		2		8		
239	5	L						5			
240	11	T			4			7			
241	8	Y		3		1			4		
242	4	V		2			1	1			
243	10	Q		4			3		2	1	
252	4	F								4	
256	25	L	1	6			5	5	3	1	4
259	9	T		2					4	2	1
260	12	I	2				1	2	2	1	4
262	1	T						1			
263	15	S				4		8	3		
264	1	G				1					
265	28	K	4	4					11	3	6
267	8	S						2			6
268	32	N				1	1			18	12
269	26	T		1	3	3	2	4	4	5	4
270	49	T		2	19	2	4	10	10	1	1
271	31	G	2	5	3	3	3	3	3	3	6
272	65	K	8	14	10	7	5		16	5	
273	67	L	10	1	10	12	11	8	3	9	3
274	46	I	8	10	5	2	9		6	5	1
275	163	W	6	26		3	14	29	10	54	21
276	46	K	5	3		1	3	6	6	15	7
277	15	V		5			4	2	1	1	2
278	29	N	5	2			3	3	4	6	6
280	14	E							6	8	
281	6	I	2	2					2		
282	4	D							4		

Table S4. Number of glycan cap antibody contacts with GP, related to Figure 3. Glycan cap antibody contacts on EBOV/BDBV GP determined by UCSF Chimera software with a default cutoff value of -0.4 Å and an allowance of 0 Å. EBOV-548 from PDB 6UYE, 13C6 from PDB 5KEL. Gradient is green-to-red, with green being fewer contacts and red being the most contacts.

Data Collection	
Resolution[a] (Å)	50.00 - 3.00 (3.05 - 3.00)
Space group	$P6_1$
Unit cell (Å)<br>(°)	94.46 94.46 94.911<br>90 90 120
Total Reflections[a]<br><br>Unique Reflections[a]<br><br>Reflections used in Refinement[a]	1103684<br><br>9472<br><br>9473
Multiplicity[a]	4.7 (4.5)
Completeness[a] (%)	97.8 (99.2)
I/σ(I)[a]	8.6 (1.4)
R_{meas}[a,b]	0.19 (1.38)
R_{pim}[a,c]	0.083 (0.612)
CC 1/2[d]	-0.295
Wilson B (Å^2)	68.35
**Refinement**	
R_{work}[e] (%)<br>R_{free}[f] (%)	0.22<br>0.27
RMSD[g] (bonds) (Å)	0.002
RMSD[g] (angles) (°)	0.51
Ramachandran favored (%)<br>Ramachandran outliers (%)	94.8<br>0
Average B-factor (Å^2)<br>Macromolecules<br>PEG	85.4<br>85.5<br>53.9

Table S5. Crystallographic Data Collection and Refinement Statistics for BDBV-289 Fab, related to Figure 4.

[a] Values in parentheses are for the highest-resolution shell.

[b] $R_{meas} = \Sigma_{hkl}\{N(hkl)/[N(hkl) - 1]\}^{1/2} \times \Sigma_i|I_i(hkl) - \langle I(hkl)\rangle| / \Sigma_{hkl}\Sigma_i I_i(hkl)$

[c] $R_{pim} = \Sigma_{hkl}\{1/[N(hkl) - 1]\}^{1/2} \times \Sigma_i|I_i(hkl) - \langle I(hkl)\rangle| / \Sigma_{hkl}\Sigma_i I_i(hkl)$

[d] $CC_{1/2} = \Sigma(x - \langle x\rangle)(y - \langle x\rangle)/[\Sigma(x - \langle x\rangle)^2\Sigma(y - \langle y\rangle)^2]^{1/2}$

[e] $R_{work} = (\Sigma_{hkl}||F_{obs}| - k\,|F_{calc}||)/(\Sigma_{hkl}\,|F_{obs}|)$.

[f] R_{free} is the same as R_{work} with 5% of reflections chosen at random and omitted from refinement.

[g] RMSD, root mean square deviation.
